# Genome-Wide RNAi Screen Identifies Broadly-Acting Host Factors That Inhibit Arbovirus Infection

**DOI:** 10.1371/journal.ppat.1003914

**Published:** 2014-02-13

**Authors:** Ari Yasunaga, Sheri L. Hanna, Jianqing Li, Hyelim Cho, Patrick P. Rose, Anna Spiridigliozzi, Beth Gold, Michael S. Diamond, Sara Cherry

**Affiliations:** 1 Department of Microbiology, University of Pennsylvania School of Medicine, Philadelphia, Pennsylvania, United States of America; 2 Penn Genome Frontiers Institute, University of Pennsylvania, Philadelphia, Pennsylvania, United States of America; 3 Departments of Medicine, Molecular Microbiology, Pathology & Immunology, Washington University School of Medicine, St Louis, Missouri, United States of America; Massachusetts General Hospital, United States of America

## Abstract

Vector-borne viruses are an important class of emerging and re-emerging pathogens; thus, an improved understanding of the cellular factors that modulate infection in their respective vertebrate and insect hosts may aid control efforts. In particular, cell-intrinsic antiviral pathways restrict vector-borne viruses including the type I interferon response in vertebrates and the RNA interference (RNAi) pathway in insects. However, it is likely that additional cell-intrinsic mechanisms exist to limit these viruses. Since insects rely on innate immune mechanisms to inhibit virus infections, we used *Drosophila* as a model insect to identify cellular factors that restrict West Nile virus (WNV), a flavivirus with a broad and expanding geographical host range. Our genome-wide RNAi screen identified 50 genes that inhibited WNV infection. Further screening revealed that 17 of these genes were antiviral against additional flaviviruses, and seven of these were antiviral against other vector-borne viruses, expanding our knowledge of invertebrate cell-intrinsic immunity. Investigation of two newly identified factors that restrict diverse viruses, dXPO1 and dRUVBL1, in the Tip60 complex, demonstrated they contributed to antiviral defense at the organismal level in adult flies, in mosquito cells, and in mammalian cells. These data suggest the existence of broadly acting and functionally conserved antiviral genes and pathways that restrict virus infections in evolutionarily divergent hosts.

## Author Summary

West Nile virus (WNV) is an insect-borne virus that has re-emerged globally and for which there are no specific therapeutics or vaccines. We set out to identify cellular factors that impact infection using Drosophila as a model insect. Using a genome-wide RNAi screen we identified a large number of genes that altered WNV infection. We focused on genes that restricted infection and validated 50 genes that were conserved from insects to humans that inhibited infection. Since WNV is a flavivirus, we tested whether additional flaviviruses were restricted by these genes and found that 17 also had antiviral activity against Dengue virus. There are additional families of insect-transmitted viruses that infect humans. Accordingly, we tested whether these genes also were antiviral against the bunyavirus Rift Valley Fever virus, the alphavirus Sindbis virus and the rhabdovirus Vesicular Stomatitis virus. From this analysis, we identified seven genes that are antiviral against all of these divergent arthropod-borne pathogens expanding our knowledge of cell-intrinsic immunity in insects. Lastly, we found that XPO1 and the Tip60 complex had antiviral activity in mammalian cells. These data demonstrate the existence of previously unknown antiviral genes that restrict infection of multiple viruses across divergent hosts.

## Introduction

Historically, West Nile virus (WNV) epidemics were observed in Africa, the Middle East, Europe, India, Australia, and parts of Asia, In 1999, WNV entered into the North America as part of an outbreak of neuroinvasive disease in New York City [1], and since then has become endemic in the United States with large numbers of cases occurring annually in different regions of the country. Indeed, the occurrence, size, and severity of outbreaks in humans overall have increased worldwide since the mid 1990s [2], with a large outbreak in Texas in 2012 leading to many fatalities [3], [4]. Different strains of WNV, with variable worldwide distributions, exhibit significant differences in pathogenesis. In humans infected with North American WNV strains, approximately 80% of infections are asymptomatic, with 20% developing WNV fever and other relatively mild symptoms, and 1% progressing to encephalitis, meningitis, or flaccid paralysis [2]. In contrast, WNV-Kunjin, endemic in Australia, has not been associated with any human fatalities or severe disease [5]. The natural transmission cycle of WNV is between mosquitoes and birds, with humans, horses, and other vertebrates being incidental dead-end hosts [2]. WNV is a member of the Flavivirus genus, which includes many globally important vector-borne pathogens, such as Dengue (DENV), yellow fever (YFV), tick-borne encephalitis (TBEV), and Japanese encephalitis viruses (JEV) [6]. DENV is endemic in more than 110 countries with 3.6 billion people at risk, and 390 million people infected yearly [7], [8]. At present, there are no specific antiviral therapies against any flavivirus, and only three insect-borne flaviviruses have approved vaccines for humans (YFV, TBEV, and JEV) [9].

Flaviviruses are small (∼50 nm diameter) enveloped viruses that contain a single-stranded, positive-sense RNA genome of ∼11-kb with a 5′ cap, but unlike mRNA, lack a 3′ polyadenylated tail [10]. WNV enters both vertebrate and invertebrate cells through clathrin-mediated endocytosis [11], and then traffics to an acidic compartment that facilitates viral fusion with endosomal membranes and release of the nucleocapsid into the cytoplasm [12]. The viral genome encodes one open reading frame and is translated as a single polyprotein at the rough endoplasmic reticulum (ER), which is subsequently processed by both viral and cellular proteases into 3 structural and 7 non-structural viral proteins [13]. Viral RNA replication occurs within cytoplasmic complexes associated with perinuclear membranes requiring lipid rearrangements [14], [15], [16], [17], and progeny viruses bud into the ER and traffic through the Golgi network where virions are processed into mature particles prior to exocytosis [18].

While there has been extensive study into the cellular pathways that are hijacked to facilitate WNV infection in mammalian cells, less is known about the cell-intrinsic pathways that restrict WNV in insects and whether these pathways have conserved roles in vertebrates. Furthermore, Flaviviruses belong to a larger group of vector-borne RNA viruses (including Togaviruses and Bunyaviruses), raising the possibility that these viruses as a group may be restricted using shared host defense pathways. Indeed, RNA interference (RNAi) is recognized as a major antiviral mechanism in insects and is active against all human arthropod-borne viruses tested including the flaviviruses WNV and DENV [19], [20]. The Jak-STAT and Toll pathways also are active in diverse insect hosts and restrict flavivirus infection in mosquitoes [19], [20]. Indeed, many antiviral pathways active in vector insects were first shown to restrict viral infection in the fruit fly (*Drosophila melanogaster*) model. This is in part due to the depth of *Drosophila* genome annotation, powerful genetic tools, potent gene silencing by RNAi, limited genetic redundancy, a high percentage of identifiable functional orthologs in both mosquitoes and vertebrates, lack of an acquired immune system, and that *Drosophila* can be experimentally infected by a large number of human arthropod-transmitted viruses. Furthermore, RNAi screening is robust in *Drosophila* cells and has been used effectively to analyze host-pathogen interactions and identify genes involved in antiviral defense including components of the RNAi silencing machinery [21], [22], [23], [24]. Additionally, findings in *Drosophila* have been extended to mosquitoes and mammals further validating this approach [23], [24], [25], [26], [27], [28], [29], [30], [31].

In this study, we used Drosophila to identify cell-intrinsic antiviral genes that restrict WNV and hypothesized that a number of these would restrict other insect-borne viruses, and some might have conserved roles in vector insects such as mosquitoes. Since many antiviral pathways (e.g., autophagy, Jak/Stat and Toll pathways) are active both in mammals and insects, we speculated that some of these newly identified factors also would confer antiviral activity in mammalian cells. To identify such genes using an unbiased approach we performed a genome-wide high-content RNAi screen in *Drosophila* cells to identify cellular factors that limited WNV infection. To date, RNAi screens have mainly focused on cellular factors usurped by pathogens to promote infection. While 22 restriction factors have been identified as anti-flaviviral in genome-wide RNAi screens [24], [32], only 2 of these are conserved between humans and insects. We optimized the assay for the discovery of restriction factors and identified 50 genes that when silenced resulted in enhanced WNV infection in *Drosophila* cells. All 50 are conserved in mosquitoes and 86% have clearly defined human orthologs. Furthermore, 17 of these genes had antiviral activity against multiple flaviviruses, and 7 genes were antiviral against a diverse panel of additional vector-borne RNA viruses. We focused on two broadly acting conserved genes, dRUVBL1 (pontin) and dXPO1 (embargoed), and found both restricted viral infection in adult flies, were antiviral in mosquito *Aedes aegypti* cell culture as well as in human cells. Furthermore, since WNV is neurotropic we tested whether RUVBL1 contributes to control of WNV in neurons and found it to be antiviral in these cells. Mechanistically, our studies establish that dRUVBL1 along with other members of the Tip60 histone acetylase complex are antiviral suggesting a role for this complex in virus restriction. Furthermore, we found that dXPO1 controls the nuclear export of specific host mRNAs, including the mRNA encoding Aldolase, which we identified as antiviral. Collectively, we identified additional novel, broadly acting cell-intrinsic antiviral genes in *Drosophila* at least some of which function in mosquito and vertebrate cells.

## Results

### RNAi screening of WNV infection of *Drosophila* cells

To identify cellular factors that restrict WNV infection, we first characterized the infection of a pathogenic North American WNV isolate (New York 2000) (referred to as WNV) [33], in *Drosophila* DL1 cells. WNV successfully infected and produced infectious virions from DL1 cells, although infection levels were substantially lower than that observed in human cells (**Figure S1A and B in Text S1**). Kinetic experiments revealed that peak immunofluorescence signal of virally produced NS1 protein was 48 hours post infection (hpi), a time point prior to substantial virus spread (**Figure S1B and C in Text S1**). We next tested whether WNV infection of *Drosophila* cells was dependent on similar entry and replication pathways as in mammalian and mosquito cells. Chlorpromazine, an inhibitor of clathrin-mediated endocytosis, blocks entry of WNV in both mammalian and mosquito cells [34], [35], and also effectively inhibited WNV infection of *Drosophila* DL1 cells (**Figure S1D in Text S1**). Ribavirin, a nucleoside analog and a inhibitor of Flavivirus replication in many mammalian cell types [36], also inhibited WNV infection of *Drosophila* cells (**Figure S1E in Text S1**).

**Figure 1:**
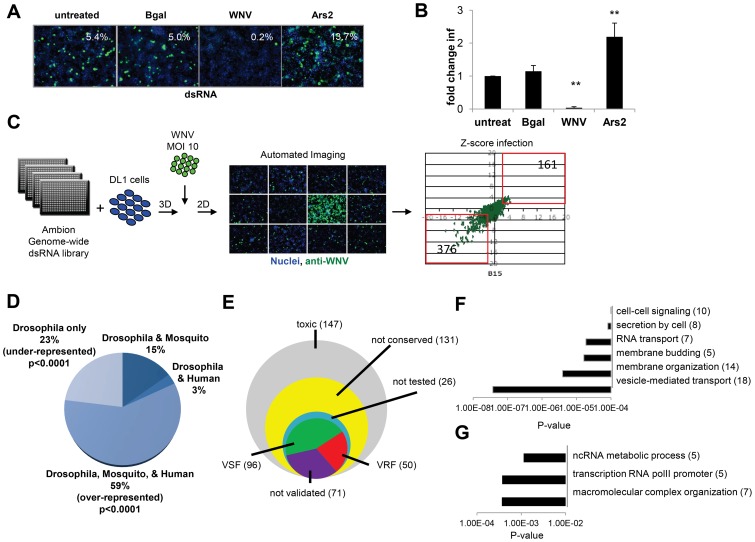
Genome-wide RNAi screen in *Drosophila* for host factors involved in WNV infection. **A.** Representative images of DL1 cells treated with the indicated dsRNAs and infected with WNV (nuclei, blue; WNV NS1, green). **B**. Quantification of fold change in infection for dsRNA treated cells as in A. Mean ± SD for 3 independent experiments; ** p<0.01. **C**. Schematic of screening pipeline including the scatter plot of Robust Z-scores for each gene assayed in duplicate. VSFs (376) and VRFs (161) are noted. **D**. Bioinformatics show fraction of candidate genes that have human or mosquito orthologs. Significant enrichment of conserved genes (p<0.0001) and under-enrichment of *Drosophila*-specific genes (p<0.0001) as analyzed by chi-squared test. **E**. Pie chart of candidate genes and validation results (50 VRF, 96 VSF, 71 not validated). **F–G**. Gene ontology enrichment of validated genes with five or more members displayed (p<0.001). **F**. VSF categories enriched. **G**. VRF categories enriched.

Next, we optimized RNAi in a 384-well format using dsRNAs against β-galactosidase (βgal) as a negative control, and dsRNA against the WNV genome as a positive control (**Figure 1A and B**). We also included dsRNA targeting Ars2, a gene that we previously established as antiviral in *Drosophila* against many unrelated RNA viruses [21]. By selecting the infection level at ∼7%, this maximized the fold-change in infection upon loss of Ars2, allowing us to focus the assay on genes which restrict infection. This approach contrasts with previous screens that used a higher infection level and focused on genes that promote infection [24], [32]. Briefly, DL1 cells were seeded onto 384 well plates pre-arrayed with dsRNAs, incubated for 3 days for effective knockdown of target genes, and infected with WNV (Multiplicity of infection (MOI) of 10) for 48 hours. Cells were fixed, permeabilized and stained for the viral protein NS1 [37] and counterstained for nuclei. Automated microscopy and image analysis calculated the cell number per well (nuclei) and number of infected cells (WNV NS1) to measure the percent infection. As expected, we observed a decrease in infection after treatment with dsRNA against WNV. Importantly, we also observed a robust increase in WNV infection upon loss of Ars2 (**Figure 1A and B**); thus these optimized conditions were used for RNAi screening.

A genome-wide RNAi screen was performed in duplicate and statistical analysis identified 537 genes (3.6% of the *Drosophila* genome) that when silenced had a significant effect on the percentage of WNV infected cells, with a robust Z score of ≥2 or ≤−2 in both replicates (p<0.001; ∼40% change; **Figure 1C**). None of the non-targeting controls spotted on each plate were identified whereas 100% of the positive control dsRNAs spotted on each plate against WNV genome and Ars2 were identified. Silencing of 376 of these 537 genes resulted in decreased viral infection, indicating WNV was dependent on these genes for replication (viral sensitivity factors (VSF)). Silencing of 161 genes resulted in increased WNV infection suggesting they normally restrict replication (viral resistance factors (VRF)). As WNV infects mosquitoes, birds, and vertebrates we were interested in those genes having orthologs in hosts that normally encounter the virus, rather than genes annotated as *Drosophila* specific, as flies are not natural hosts. Analysis of this candidate gene list revealed that ∼59% of the genes have orthologs in both humans and mosquitoes (p<0.0001), with *Drosophila*-specific genes being greatly under-represented (∼23% of the total; p<0.0001) (**Figure 1D**). Of the 537 genes identified in the primary screen, 147 were cytotoxic (robust Z score<−2 in duplicate; ∼15% decrease in cell number) and were excluded from further analysis. Only one gene had a robust Z score>2 in duplicate but did not validate subsequently. Additionally, 131 genes were not clearly conserved in mosquitoes or humans (as determined by Homologene) and also were excluded from further analysis. Of the 280 remaining genes, we set out to validate all of the genes except for a handful that were members of complexes in which we identified >2 components. In those cases, we chose to validate representative genes from these complexes (**Table S1**). To do this, we generated independent dsRNA reagents that targeted 217 genes and screened this secondary gene set under two conditions: we infected cells at a low level of infection (4%) to maximize identification of genes that restricted infection, and at a higher level of infection (18%) to maximize validation of genes that promote infection. Of the 217 genes, 121 validated (56%): 82 genes (68%) facilitated WNV infection (VSFs) and 39 genes (32%) restricted infection (VRFs). We also validated a total of 23 genes from larger complexes (**Table S1, Table S2, and Figure S1F in Text S1**). If we include the remaining 17 genes in the complex, the screen identified 96 VSFs and 50 VRFs in total (**Figure 1E** **; Table S1 and S2**).

Bioinformatics analysis was used to identify processes or pathways that impact WNV infection. First, we performed Gene Ontology enrichment analysis on the VSF and VRF gene sets independently (**Figure 1F and G**) and found biological pathways including vesicle-mediated transport and membrane modifications were enriched within the VSF data set, consistent with the important role of vesicular trafficking and membrane modifications in WNV entry and replication [6]. Second, we used several functional annotation metrics to place these validated genes into cellular pathways and sub-cellular compartments most likely relevant to WNV infection (red genes, VRF; green genes, VSF; black genes not tested but in validated complexes; **Figure S1G in Text S1**). We identified 29 genes involved in endocytosis and endosomal acidification, a known entry pathway for WNV. Furthermore, although we tested and validated only 4 of the components in the signal recognition particle complex, we identified 6 subunits of this complex in our primary screen, supporting the importance of targeting the WNV polyprotein to the ER for proper translation and processing. These findings suggest that this screen was robust and identified important host factors that promote infection.

The VRFs were highly conserved (86% have human orthologs) and fell into distinct enriched groups. Two of the three categories involved RNA metabolism, including RNA transcription, which may be involved in an antiviral transcriptional program [31]. In fact, 28% of the WNV VRFs (p<0.00012) have a function within the nucleus suggesting a complex host response to infection since WNV replicates exclusively in the cytoplasm (**Figure S1F in Text S1**).

### Identification of broadly antiviral factors in vector-borne virus infections

**Figure 2:**
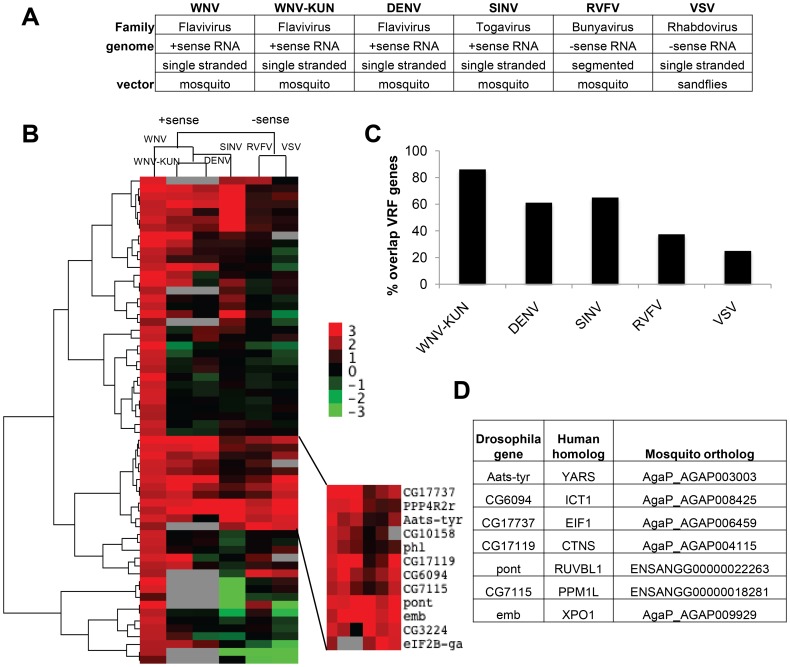
Comparisons of virus dependencies. **A.** Table listing the vector-borne viruses tested and general classifications based on virus family, genome structure, and natural vector. **B**. A hierarchical heat map of six viruses screened displaying Robust Z-scores for each VRF against WNV, WNV-KUN, DEN, SINV, RVFV, and VSV. **C**. Percentage of WNV VRFs that also restricted the indicated virus. **D**. Table of the seven *Drosophila* genes that had antiviral activity against all six viruses tested. Human and mosquito orthologs are listed.

While few antiviral pathways have been described in *Drosophila*, the well characterized ones (e.g., RNA silencing machinery) appear to inhibit infection of diverse viruses [38], [39]. Given this, we explored whether the anti-WNV factors identified also restricted other viral pathogens. We tested two additional flaviviruses: the WNV strain Kunjin (CH 16532; WNV-KUN) and Dengue virus (*Drosophila* adapted Dengue-2 (DENV)). In addition we tested three additional human vector-borne viruses: Sindbis virus (HRsp; SINV), Rift Valley Fever virus (MP12; RVFV), and vesicular stomatitis virus (Indiana; VSV) (**Figure 2A**). All of these are enveloped RNA viruses transmitted to vertebrates by an insect vector. While mosquitoes are the natural vector for WNV, WNV-KUN, DENV, SINV and RVFV, sandflies are the primary vector for VSV. The flaviviruses and SINV are positive sense RNA viruses, whereas RVFV and VSV are negative sense. RVFV has a tri-segmented genome, while the other viruses encode a non-segmented genome. Thus, these viruses represent divergent families and genomic architectures.

We and others have previously infected *Drosophila* with WNV, DENV, SINV, RVFV and VSV [24], [25], [40], [41], [42]. However, WNV-KUN infection of *Drosophila* has not been characterized. WNV-KUN is a less pathogenic strain of WNV endemic to Oceania [5]. We found that WNV-KUN, analogous to WNV New York, productively infected *Drosophila* cells (**Figure S2A and B in Text S1**). Next, we optimized conditions for RNAi screening in 384 well plates using both negative and positive control dsRNAs based upon our previous studies selecting conditions to identify restriction factors for WNV-KUN, DENV, SINV, RVFV and VSV (**Figure S2C–G in Text S1**) [25], [40], [41]. We screened the validated WNV gene set in duplicate against each virus, and Z-scores were calculated (**Table S3**). We used hierarchical clustering to compare the VRF gene dependencies of all six viruses (**Figure 2B**). The four positive sense viruses clustered together (flaviviruses WNV, WNV-KUN, and DENV, and alphavirus SINV), while RVFV and VSV, the two negative sense viruses clustered together. This suggests the gene signature of restriction is related to a fundamental aspect of viral structure.

The WNV VRFs had a high propensity to impact infection by multiple different viruses. There was a high concordance of gene dependencies across the three flaviviruses; 31 genes (86%) restricted WNV-KUN and 22 genes (61%) restricted DENV (**Figure 2C** **and Table S3**). There also was a large overlap between WNV and SINV VRFs (64%), while less so with RVFV (38%) and VSV (25%). Thus, many anti-WNV factors appear broadly antiviral against other flaviviruses and an unrelated positive strand RNA virus in insect cells. The degree of VRF overlap diminished as the viruses became more disparate (**Figure 2C**). Nonetheless, we identified 7 host factors that significantly restricted infection by all six vector-borne viruses tested (p<0.05): dXPO1 (emb), dRUVBL1 (pont), dYARS (Aats-tyr), dEIF1B (CG17737), dPPM1L (CG7115), dCTNS (CG17119) and dICT1 (CG6094). All seven of these VRF genes have human and mosquito orthologs (**Figure 2D**).

### dRUVBL1 and the Tip60 complex restrict vector-borne virus infection in *Drosophila*

**Figure 3:**
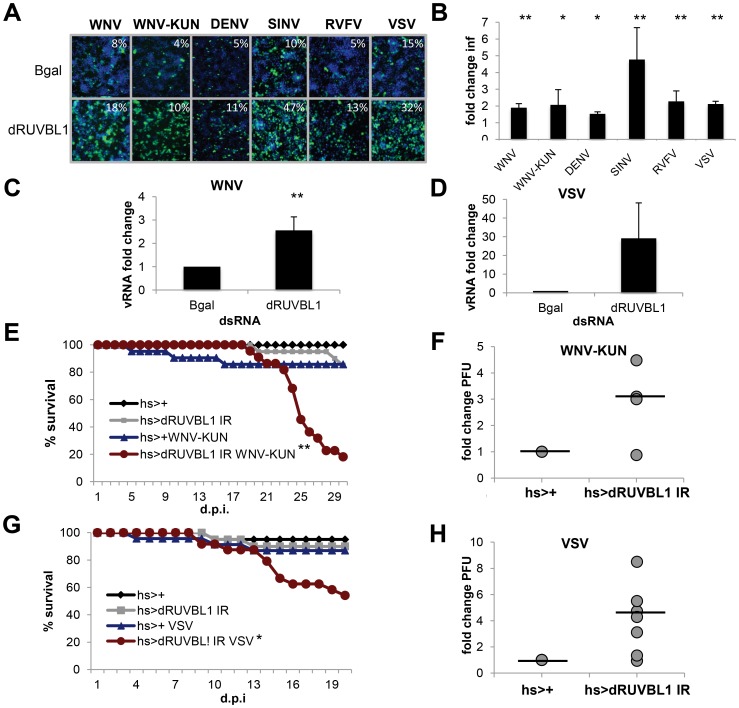
dRUVBL1 is a broadly antiviral gene. **A.** Representative images of *Drosophila* cells treated with control (β-gal) or dRUVBL1 dsRNA, and infected with WNV, WNV-KUN, DEN, SIN, RVFV, or VSV (blue, nuclei; green, virus). **B**, Quantification of fold change in infection for dsRNA treated cells as in A. Mean ± SD for 3 independent experiments; * p<0.05, ** p<0.01. **C–D**. Viral RNA levels measured using qRT-PCR in *Drosophila* cells treated with β-gal (control) or dRUVBL1 dsRNA infected with WNV (**C**) or VSV (**D**) Mean ± SD of fold change for 3 independent experiments; ** p<0.01. **E–H**. Adult flies of the indicated genotypes were challenged with vehicle or WNV-KUN (**E–F**) or VSV (**G–H**). Mortality was monitored as a function of time post-infection (**E,G)** (log rank: * p<0.05, ** p<0.01). (**F,H)** Groups of 15 flies of the indicated genotypes were challenged, and viral titers were assessed by plaque assay in 4–7 independent experiments (shown as individual dots) with controls (set to 1) and fold change shown at day 6 post infection. Line represents mean.

Among the validated WNV VRFs, genes with putative nuclear roles were enriched (p<0.00012) and included dRUVBL1 (pontin, also known as Tip49), which was antiviral against all six viruses. RUVBL1 is an ATP-binding protein belonging to the AAA+ (ATPase associated with diverse cellular activities) family of ATPases implicated in diverse cellular pathways in the nucleus and cytoplasm [43], [44], [45], [46], [47], [48], [49]. First, we validated the antiviral activity of dRUVBL1 using independent dsRNA targeting dRUVBL1 outside of the screening format and observed a significant increase (p<0.05) in infection by WNV, WNV-KUN, DENV, SINV, RVFV and VSV compared to control (**Figure 3A and B**). There was no impact on cell number upon depletion of dRUVBL1 (**Figure S3A in Text S1**). Next, using quantitative RT-PCR (RT-qPCR) as an independent assay, we found that both WNV and VSV RNA levels were increased upon dRUVBL1-depletion compared to the control (**Figure 3C and D**).

One advantage of the *Drosophila* system is the powerful genetic tools including the availability of genome-wide *in vivo* RNAi transgenic flies. Furthermore, *Drosophila* are not hematophagous, so they can be challenged easily and safely with highly pathogenic human viruses. We took advantage of WNV-KUN as it is a BSL2 agent in comparison to the more virulent North American WNV strains, which require a BSL3 facility [50]. Wild-type flies were permissive to WNV-KUN infection as measured by plaque assay and exhibited no increase in mortality compared to control flies (**Figure S3B and C in Text S1**). This is consistent with the natural infection of mosquitoes where limited pathogenesis is observed, and similar to our observations with other vector-borne viruses (VSV, SINV, RVFV) that display limited pathology upon viral infection [25], [40], [41]. However, loss of innate immune defenses in *Drosophila* or mosquitoes can render insects more susceptible to infection and result in increased viral replication and mortality [19], [20], [21], [40], [51], [52], [53]. Because null mutants in dRUVBL1 are lethal, we took advantage of inducible RNAi transgenic flies [54]. We used the GAL4/UAS system to promote expression of a UAS- inverted repeat (IR) transgene that bears long hairpin dsRNA against dRUVBL1 to target the endogenous transcript *in vivo*. We induced expression of the transgene using a heat shock (hs) promoter in adult flies allowing us to bypass any developmental requirements. Indeed, expression of the hairpin during development was lethal (data not shown). Importantly, heat shock driven dRUVBL1 RNAi flies had decreased dRUVBL1 mRNA (**Figure S3D in Text S1**). Next, dRUVBL1-depleted (hs-GAL4<dRUVBL1 IR) and control flies (hs-GAL4<+) were challenged with WNV-KUN and survival was monitored. Unchallenged dRUVBL1-depleted flies exhibited no increase in mortality nor did control flies challenged with WNV-KUN. Notably, the majority of WNV-KUN infected dRUVBL1-depleted flies succumbed to infection (p<0.01, **Figure 3E**). We next tested if there was an impact on viral load. Groups of 15 flies were challenged, and whole animals were crushed, and assayed for WNV-KUN by plaque assay in four independent experiments (shown as individual dots). We observed modest, but increased viral loads in dRUVBL1-depleted animals compared to controls (set to 1) at day 6 post infection; similar results were observed at day 9 post infection (**Figure 3F**, not shown).

We subsequently explored the requirement of dRUVBL1 during VSV infection, the best-studied human arbovirus in flies, and most divergent from WNV of the vector-borne viruses tested (**Figure 2C**). Again, while uninfected flies or wild type control flies challenged with VSV exhibited little mortality, flies depleted for dRUVBL1 and challenged with VSV showed an increase in mortality after infection (p<0.01, **Figure 3G**). Groups of 15 flies were challenged, and whole animals were crushed, and assayed for VSV by plaque assay in seven independent experiments (shown as individual dots). We observed modest, but increased viral loads in dRUVBL1-depleted animals compared to controls (set to 1) at day 6 post infection (**Figure 3H**). Together, these results demonstrate the important and broad-spectrum antiviral requirement for dRUVBL1 both *in vitro and in vivo* in *Drosophila*.

**Figure 4:**
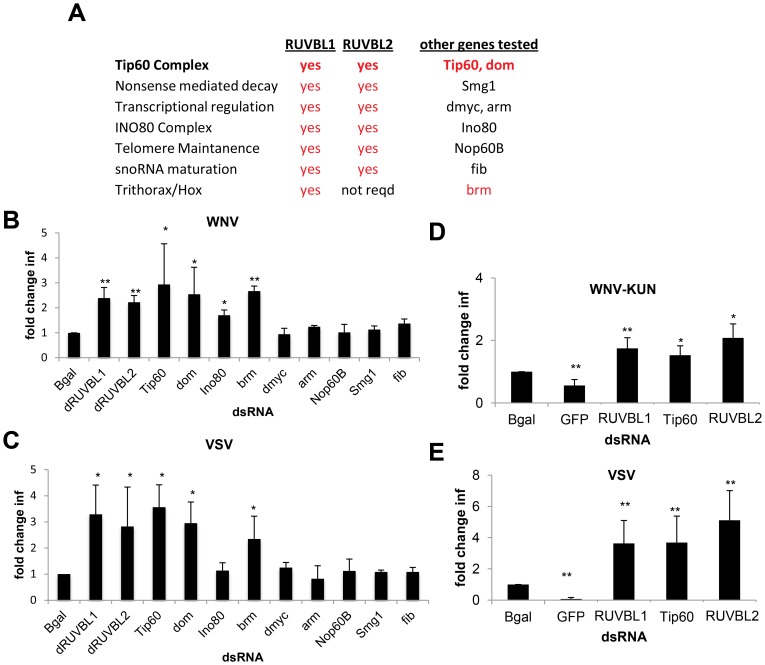
Tip60 complex has antiviral activity. **A.** Table of RUVBL1-associated complexes, whether the complex is dependent on RUVBL2, and other genes in the complexes tested for antiviral activity. Genes in red were found to be antiviral against both WNV and VSV. **B–C**. DL1 cells were treated with the indicated dsRNA and then infected with (**B**) WNV or (**C**) VSV. Mean ± SD of fold change in percent infection compared to control (bgal dsRNA) for 3 independent experiments; * p<0.05, ** p<0.01. **D–E**. Aag2 cells were treated with the indicated dsRNA and then infected with (**D**) WNV-KUN or (**E**) VSV. Mean ± SD of fold change in percent infection compared to control (bgal dsRNA) for 3 independent experiments; * p<0.05, ** p<0.01.

dRUVBL1 has been shown to function in many complexes, most often in conjunction with another AAA+ ATPase, dRUVBL2 (reptin, also known as Tip48) (depicted in **Figure 4A**) [43], [54]. Indeed, structural and functional analysis of human and yeast RUVBL1 and RUVBL2 suggest these proteins work as a scaffold in addition to functioning as ATPases, potentially explaining their association with a diverse set of cellular complexes [44]. dRUVBL1, along with dRUVBL2, is involved in chromatin remodeling, most notably in the Ino80 and Tip60/Swr1 complexes [55], [56]. Furthermore, roles in transcriptional regulation facilitating the activity of c-Myc and β-catenin also have been reported in *Drosophila* and human cells [57], [58]. Additional roles for dRUVBL1 and dRUVBL2 have been described in snoRNA maturation, nonsense mediated mRNA decay, and telomere maintenance. Lastly, dRUVBL1 also has been implicated in chromatin remodeling with the *Drosophila* Trithorax complex, although this is thought to be independent of dRUVBL2 [46]. Based on these possible functions, we tested components of these complexes for their impact on viral infection to identify which of the putative dRUVBL1 containing complex(es) mediated the antiviral activity. We designed dsRNAs against dRUVBL2 along with the indicated genes in each of the complexes **in** **Figure 4A**. Cells were treated with these dsRNAs, along with β-gal (negative) and dRUVBL1 (positive) controls. Importantly, no impact on cell viability was observed (**Figure S4A in Text S1**). Next, the dsRNA treated cells were infected with either WNV or VSV. Depletion of c-Myc, arm (*Drosophila* β–catenin), Smg1, Fib and Nop60B did not impact WNV or VSV infection levels (**Figure 4B and C**). In contrast, dRUVBL2 was antiviral against both VSV and WNV (**Figure 4B and C**). Depletion of both dRUVBL1 and dRUVBL2 together did not increase infection beyond that observed with silencing of either gene, indicating their effect was not additive (data not shown). Increased WNV and VSV infection also was observed when dTIP60 (Tip60), dEP400 (domino (dom)) and dSMARCA4 (Brahma (brm)) were depleted. Since dRUVBL1, dRUVBL2, dEP400 and dTIP60 are all antiviral, and members of the Tip60 complex, these data suggest that a major antiviral role of dRUVBL1 is through its function in the Tip60 complex.

Next, we tested whether Tip60 also restricted infection of adult flies. Indeed, we depletion of Tip60 using *in vivo* RNAi led to decreased survival of flies challenged with WNV-KUN and VSV but did not impact survival of unchallenged animals (**Figure S4E–G in Text S1**). Thus, the Tip60 complex also has antiviral roles *in vivo*.

### Tip60 complex is antiviral in mosquito cells

Mosquitoes are the natural vectors for WNV, WNV-KUN, DENV, RVFV and SINV although the particular mosquito species that transmit each of these viruses varies [59], [60]. In contrast, the primary vector for VSV is the sandfly, although VSV has been isolated from mosquitoes [61]. *Aedes aegypti* is the primary vector species for DENV transmission, and can be infected by RVFV, WNV, WNV-KUN, and SINV [62]. Furthermore, the *Aedes aegypti* genome has been sequenced [63] and the *Aedes aegypti* cell line Aag2 is amenable to RNAi and routinely used as a model for mosquito cell studies [64].

We designed dsRNAs against *Aedes aegypti* RUVBL1 (AAEL004686), RUVBL2 (AAEL010341), and TIP60 (AAEL014072) orthologs. Prior to infection, Aag2 cells were treated with these dsRNAs or with dsRNAs against Bgal or the viral genome as negative and positive controls, respectively. Loss of RUVBL1, RUVBL2, or TIP60 mosquito orthologs did not affect cell number (**Figure S4B in Text S1**) but led to a significant increase in WNV-KUN infection (p<0.05, **Figure S4C in Text S1 and** **Figure 4D**). Similarly, each of these genes had antiviral effects against VSV, as silencing resulted in increased infection (p<0.05, **Figure S4D in Text S1 and** **Figure 4E**). These data indicate that members of the Tip60 complex also have antiviral activity in cells from a mosquito vector.

### Export receptor, dXPO1, restricts virus infection in *Drosophila*

**Figure 5:**
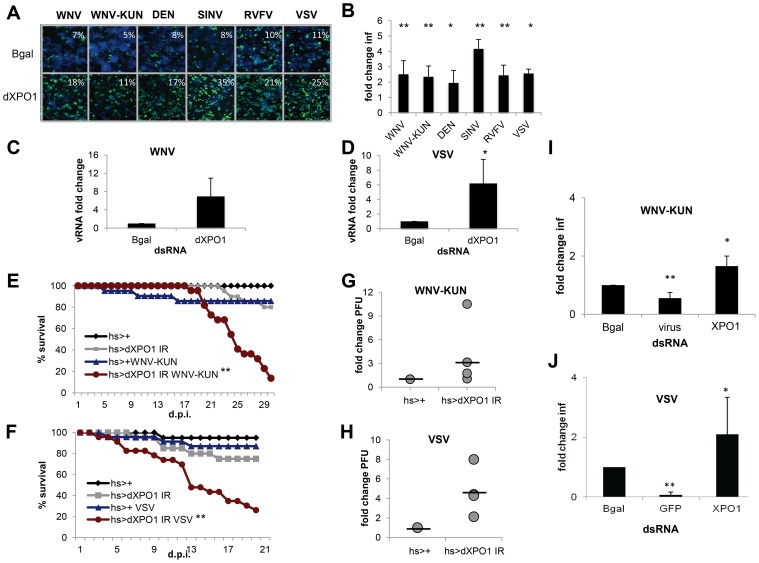
dXPO1 has antiviral activity in insects. **A.** Representative images of *Drosophila* cells treated with control (β-gal) or dXPO1 dsRNA, and infected with WNV, WNV-KUN, DEN, SIN, RVFV, or VSV (blue, nuclei; green, virus). **B**. Quantification of fold change in infection for dsRNA treated cells as in **A**. Mean ± SD for 3 independent experiments; * p<0.05, ** p<0.01. **C–D**. Viral RNA levels measured using RT-qPCR in Drosophila cells treated with β-gal (control) or dXPO1 dsRNA and infected with WNV (**C**) or VSV (**D**). Mean ± SD of fold change for 3 independent experiments; * p<0.05. **E–H**. Adult flies of the indicated genotypes were challenged with vehicle or WNV-KUN (**E, G**) or VSV (**F, H**) and mortality (**E, F**) was monitored as a function of time post-infection (** p<0.01 log rank). (**G, H**) Groups of 15 flies of the indicated genotypes were challenged, and viral titer was assessed by plaque assay in 3 or 4 independent experiments (shown as individual dots) with controls (set to 1) and fold change shown at day 6 post infection. Line represents mean. **I–J**. Aag2 cells were treated with the indicated dsRNA and then infected with (**I**) WNV-KUN or (**J**) VSV. Mean ± SD of fold change in percent infection compared to control (β-gal dsRNA) for 3 independent experiments; * p<0.05, ** p<0.01.

dXPO1 (embargoed (emb), also known as CRM1), another broadly antiviral gene identified in our screen, is a nuclear export receptor conserved from yeast to humans. XPO1 shuttles proteins and RNAs from the nucleus to the cytoplasm [65], [66]. To validate the role of dXPO1 in viral infection we tested whether an independent dsRNA against dXPO1 modulated infection. Silencing of XPO1 with an independent dsRNA did not impact cell number (**Figure S5A in Text S1**) but resulted in to 2 to 4-fold increases in the percentage of cells infected with WNV, WNV-KUN, DENV, SINV, RVFV or VSV as measured by microscopy (p<0.05, **Figure 5A and B**). Consistent with this, loss of dXPO1 led to a ≥6-fold increase in both WNV and VSV RNA, as measured by RT-qPCR (p<0.05, **Figure 5C and D**). Thus, a loss of dXPO1 expression leads to increased viral replication in *Drosophila* cells.

Next, we assessed whether dXPO1 was antiviral *in vivo* in adult flies. Null mutants of dXPO1 are lethal [67] so we again used an inducible RNAi and observed *in vivo* silencing of the mRNA (**Figure S5B in Text S1**). We then challenged control (hs-GAL4>+) or dXPO1-depleted (hs-GAL4>dXPO1 IR) flies with vehicle, WNV-KUN or VSV. While unchallenged flies or control challenged flies did not exhibit increased mortality, dXPO1-depleted flies challenged with either WNV-KUN or VSV had increased mortality (p<0.01, **Figure 5E and F**). Furthermore, dXPO1-depleted flies had modestly increased WNV-KUN viral loads, as measured by plaque assay of whole flies in four independent experiments (individual dots) relative to control (set to 1) (**Figure 5G**). And increased VSV loads, as measured by plaque assay of whole flies in three independent experiments (individual dots) relative to control (set to 1) (**Figure 5H**). These results establish that dXPO1 is required for antiviral defense both in cells and at the organismal level in adult flies.

### XPO1 is antiviral in mosquito cells

To assess whether XPO1 also had antiviral activity in the vector mosquito cells, we treated Aag2 cells with dsRNAs against the *Aedes aegypti* XPO1 ortholog (AAEL001484) or against Bgal or the viral genome as negative and positive controls, respectively. These cells were subsequently challenged with WNV-KUN or VSV. While depletion of XPO1 did not affect cell number (**Figure S5B in Text S1**), we observed a significant increase in the percentage of Aag2 cells infected with WNV-KUN or VSV (p<0.05, **Figure S5C and D in Text S1 and** **Figure 5I and J**).

### Aldolase, a target of dXPO1 nuclear export, is antiviral

**Figure 6:**
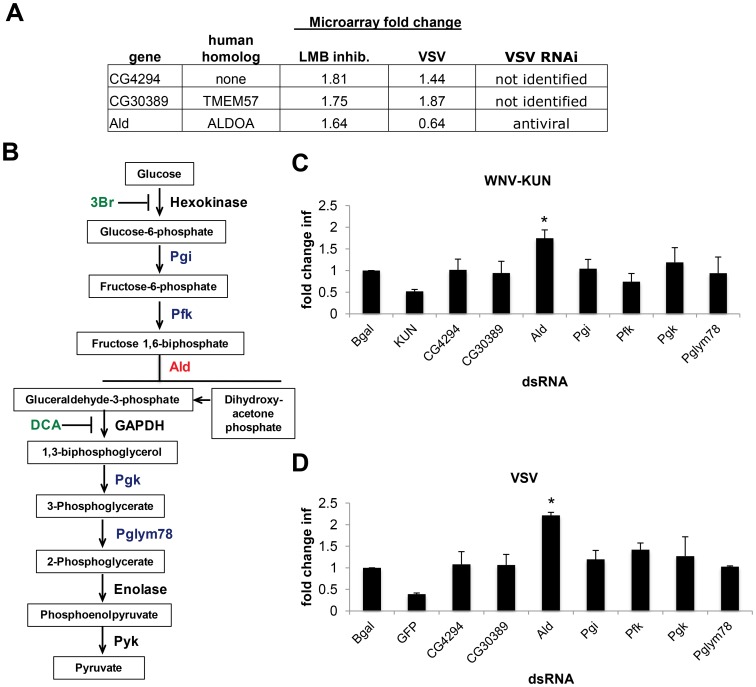
dXPO1 targets dALDOA and restricts viral infection. **A.** Table of genes whose mRNA export is LMB-dependent, their level of induction by VSV infection, and whether they have been identified as antiviral previously. **B**. Schematic overview of glycolysis pathway (red, Aldolase (Ald); blue, enzymes tested; black, enzymes not tested; green, inhibitors). **C–D**. DL1 cells were treated with the indicated dsRNA and then infected with (**C**) WNV-KUN or (**D**) VSV. Data is presented as Mean ± SD of fold change in percent infection compared to control (β-gal dsRNA) for 3 independent experiments; * p<0.05.

Since dXPO1 is as a nuclear export receptor, we speculated that dXPO1-dependent regulation of either host genes required for infection or virus-induced antiviral genes may account for the antiviral activity. Indeed, antiviral transcriptional programs have been shown to restrict viral infections in *Drosophila* [19], [31], [52], [68], [69]. Leptomycin B (LMB) is a potent and specific inhibitor of dXPO1 mediated nuclear export [70]. Previous work demonstrated that LMB treatment of *Drosophila* cells altered the nuclear export of only 85 mRNAs (<2% of the transcripts surveyed) [71]. One gene, bsg, was XPO1-dependent and required for WNV infection. However, this cannot explain the phenotype of XPO1 because bsg was required for WNV infection and not the other viruses that are sensitive to XPO1 restriction (**Table S3**). Moreover, 2 XPO1-dependent genes also were transcriptionally induced by VSV infection (CG4294, CG30389) [31]. We generated dsRNAs targeting CG4294 and CG30389 but observed no impact on WNV-KUN or VSV infection (**Figure 6C and D**). None of the 50 VRFs from our screen were within this set; however, data mining of an RNAi screen with VSV in DL1 cells (S. Cherry unpublished data) identified one additional gene (Aldolase, dALDOA) from this LMB-dependent gene set that showed antiviral activity against VSV (**Figure 6A**). We generated an independent dsRNA targeting dALDOA and observed that depletion did not affect cell number (**Figure S6A in Text S1**) but resulted in a 1.5 to 2.5-fold increase (p<0.05) in the percentage of cells infected with WNV-KUN and VSV, respectively (**Figure 6C and D**). This suggests that dXPO1-dependent mRNA export of dALDOA contributes to the defense against multiple virus families.

Aldolase is a critical enzyme in glycolysis, catalyzing the conversion of fructose 1,6-biphosphate to glyceraldehyde-3-phosphate (G3P) and dihydroxyacetone phosphate (DHAP) (**Figure 6B**). However, Aldolase may have functions apart from glycolysis, as its expression but not all core glycolytic enzymes are increased in response to LPS treatment [72]. To define whether the antiviral activity of Aldolase was related to glycolysis we performed two complementary experiments. First, we depleted *Drosophila* cells of additional enzymes essential for glycolysis (Phosphoglucose isomerase (Pgi), Phosphofructokinase (Pfk), Phosphoglycerate kinase (Pgk), and Phosphoglycerate mutase (Pglym87)) (**Figure 6B**). Depletion of these canonical glycolysis enzymes had no impact on cell number (**Figure S6A in Text S1**) or WNV-KUN and VSV infection (**Figure 6C and D**). Second, to overcome the fact that RNAi is incomplete, and that these are enzymes which may be fully active at low levels, we took advantage of two specific and potent glycolysis pathway inhibitors, Dichloracetic Acid (DCA), which inhibits the enzyme pyruvate dehydrogenase kinase, and a hexokinase inhibitor (3Br) [73]. Neither of these treatments impacted cell number (**Figure S6B–E in Text S1**) or WNV-KUN and VSV infection of *Drosophila* cells (**Figure 6B–D**). Together, these data suggest the antiviral effect of Aldolase is not mediated through the glycolysis pathway.

### XPO1 and RUVBL1 have antiviral activity in mammalian cells

**Figure 7:**
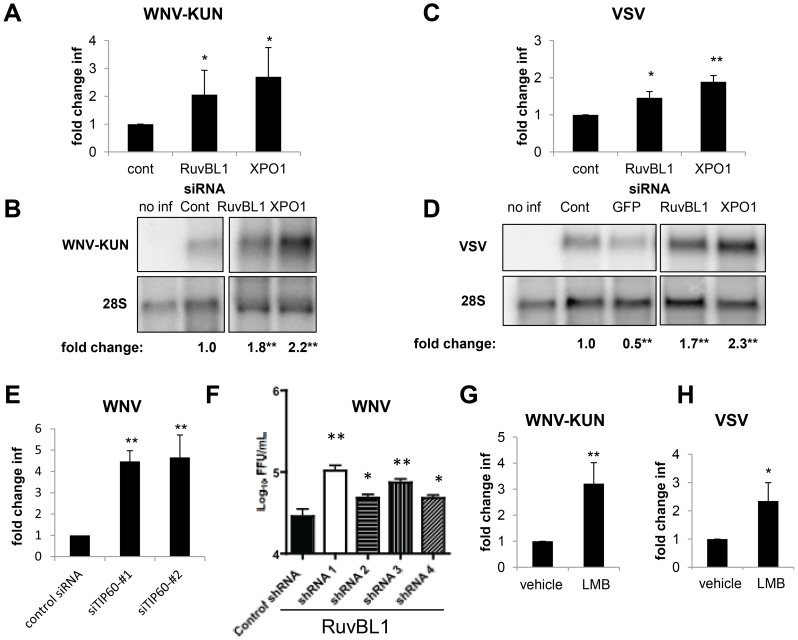
RUVBL1 and XPO1 restrict viral infection in mammalian cells. **A–D.** Human U2OS cells were transfected with siRNAs against a control, hRuvBL1, or hXPO1 and challenged 3 days post transfection with WNV-KUN for 20 hours (**A–B**) or VSV for 12 hours (**C–D**). Cells were fixed, processed for microscopy and quantified in **A, C**. Mean ± SD of fold change compared to control for 3 independent experiments; * p<0.05, **p<0.01. Cells were processed for northern blots and quantified displaying the mean for 3 independent experiments with control set to 1; * p<0.05, **p<0.01 in **B, D**. **E**. 293T cells were transfected with siRNAs against control or two independent siRNAs against hTIP60 and challenged 3 days post transfection with WNV for 24 hours and processed by flow cytometry. Three independent experiments were quantified; Mean ± SD of the fold change in infection is shown and normalized to the control; **p<0.01. **F**. Primary neurons transduced with lentiviruses expressing the indicated shRNAs were infected with WNV for 24 hours and processed for viral yield by focus forming assays. Mean ± SD for 3 independent experiments; * p<0.05, **p<0.01. **G–H**. U2OS cells were treated with vehicle or LMB and infected with (**G**) WNV-KUN or (**H**) VSV. Mean ± SD of fold change in percent infection compared to control (vehicle) for 3 independent experiments; * p<0.05, ** p<0.01.

As dRUBVL1 and dXPO1 are conserved from insects to mammals, we tested whether silencing of these genes in human cells impacted infection. For these studies, we transfected human osteosarcoma U2OS cells with siRNAs against a non-targeting control, hRUVBL1 or hXPO1. Three days later, we confirmed silencing of these genes by RT-qPCR (**Figure S7A and B in Text S1**) with no impact on cell number (**Figure S7C in Text S1**). Next, the cells were infected with WNV-KUN (MOI of 0.5), and infection levels were monitored using immunofluorescence 20 hpi. Loss of either hRUVBL1 or hXPO1 resulted in a 2 to 3-fold increase in the percentage of WNV-KUN-infected cells, as measured by microscopy (p<0.05, **Figure 7A**). Consistent with this, we observed an increase in viral RNA levels in cells depleted of hRUVBL1 or hXPO1 as measured by Northern blot and quantified (p<0.05, **Figure 7B**). Similarly, depletion of hRUVBL1 or hXPO1 enhanced VSV infection, as measured by the percentage of infected cells (p<0.05, **Figure 7C**) or levels of viral RNA (p<0.05, **Figure 7D**). Furthermore, we tested whether RUVBL1 likely acted through the same Tip60 complex as we found in *Drosophila*. To this end, we obtained independent siRNAs against hTIP60 (KAT5) and confirmed they reduced TIP60 expression in human 293T cells as measured by RT-qPCR (**Figure S7D in Text S1**). Furthermore, we observed significantly increased WNV infection in the depleted cells (**Figure 7E**). Together, these data suggest that the Tip60 complex is antiviral against multiple viruses and in disparate hosts ranging from insects to vertebrates.

Our initial studies in *Drosophila* were performed in a single round of infection suggesting that the requirements for the genes in the viral lifecycle included: entry, uncoating, translation, polyprotein processing, and RNA replication. To study the step in the viral lifecycle impacted by the Tip60 complex in mammalian cells we took advantage of a human cell line (293T) that stably maintains a subgenomic WNV replicon expressing GFP [74], [75]. If these genes restricted infection downstream of entry, but upstream of assembly, they should restrict the replication of this WNV replicon. Indeed, siRNA depletion of hRUVBL1 or hTIP60 led to increased levels of WNV replicon replication as measured by immunoblot (**Figure S7E in Text S1**). Therefore, the action of these genes is at the step of translation, polyprotein processing, or RNA replication.

Since WNV is a neurotropic virus we tested whether RUVBL1 restricted infection in primary neuronal cultures. We prepared cerebellar granule cell neurons from wild-type C57BL/6 mice and transduced them with lentiviruses expressing either a control shRNA, or 4 independent shRNAs against RUVBL1. Three days later, we challenged the cells with WNV (MOI = 0.1), and harvested virus in the supernatant 24 hours later. Notably, all four independent shRNA depleted RUVBL1 to varying extents (**Figure S7F in Text S1**), and the level of depletion correlated with a significant increase in viral titers (p<0.05, **Figure 7F**). These data demonstrate that RUVBL1 restricts WNV infection in primary neurons.

To confirm a role for hXPO1 in antiviral defense in human cells using a small molecule inhibitor to complement our RNAi studies, we treated U2OS cells with the XPO1 export inhibitor LMB and monitored WNV-KUN or VSV infection. Treatment with LMB significantly enhanced (2–3 fold) viral replication by both viruses (p<0.05, **Figure 7G and H**), as measured by an increase in the percentage of infected cells. LMB treatment did not impact cell number (**Figure S7G in Text S1**). Furthermore, siRNA-mediated depletion of hXPO1 or LMB treatment of 293T cells carrying a WNV replicon revealed that the dependence was again downstream of entry and upstream of assembly since both perturbations led to increased levels of replication (**Figure S7E and S7H in Text S1**). These data suggest that the hXPO1 has antiviral activity through the regulation of XPO1-dependent cargo export downstream of entry in evolutionarily diverse cell types from insects to mammals.

## Discussion

Genome-wide RNAi screens have been employed to identify cellular factors required by viruses to successfully infect cells as well as factors that, if left unmodulated by the virus, serve to suppress infection. In addition, this screening approach can identify pathways that regulate the expression and activity of direct antiviral factors, orchestrating a robust antiviral response. Since our goal was to identify conserved inhibitory pathways that span insects and mammals with a particular interest in those having broad antiviral activity against disparate viruses, we performed a genome-wide RNAi screen in *Drosophila* in which we deliberately set a low infection rate, thereby sensitizing our assay to detect factors that when suppressed result in higher levels of infection. This is in contrast to previous genome-wide flavivirus RNAi screens, which targeted a higher level of infection and so led to the identification of a larger number of genes that promote infection [24], [32]. Nonetheless, our screen was sufficiently sensitive and robust to enable us to identify 96 genes that promoted WNV infection. Enriched gene ontology categories included pathways such as clathrin-mediated endocytosis and endosomal acidification that are required for flavivirus entry and were identified by earlier RNAi screens.

We identified 50 restriction factors, greatly expanding the number of cell intrinsic anti-WNV factors known [32], [76], [77], [78], [79], [80]. We compared our restriction factors with previous studies (Table S4). A genome-wide siRNA screen against WNV in human cells identified 22 genes that were antiviral of which 6 had Drosophila homologs; none of which were within our validated antiviral genes [32]. A genome wide screen against hepatitis C virus, a distantly related *Flaviviridae* family member, in human cells identified 25 antiviral genes of which 12 had Drosophila orthologs; again, none of which were within our gene set [81]. Two screens querying the antiviral role of interferon stimulated genes (ISGs) against flaviviruses were recently published [80], [82]; however, none of our antiviral genes are known ISGs. The Schoggins screen identified 47 ISGs that when ectopically expressed restricted a flavivirus amongst which there were 12 homologs in Drosophila; none of which we identified as antiviral in our screen. The Li screen identified 47 ISGs that when depleted by RNAi restricted infection amongst which 13 had homologs in Drosophila; none of which were identified in our screen. None of the *Drosophila* homologs from any of these screens were within any other screen making conclusions difficult. Additional screens performed at low levels of infection may reveal additional intrinsic restriction factors.

Unexpectedly, our antiviral gene set was enriched for nuclear functions such as RNA metabolism and transcription even though WNV replicates exclusively in the cytoplasm. This observation suggested that we had uncovered pathways and processes that orchestrate an antiviral response rather than factors that interact directly with the virus. If this were the case, as is seen with antiviral interferon (IFN) responses in mammals, we reasoned that many of the VRFs might have antiviral activities against additional viruses. This in fact proved to be the case - not only did we discover a high degree of concordance between the WNV, WNV-KUN and DEN VRFs (WNV-KUN 86%, 31 genes; DENV 61%, 22 genes), we identified seven genes that restricted infection of all six different arboviruses tested, which included both positive and negative-sense RNA genomes: dYARS (Aats-tyr), dEIF1(CG17737), dPPM1L (CG7115), dCTNS (CG17119), dICT1 (CG6094), dXPO1 (emb), and dRUVBL1 (pont). Since none of these genes have been suggested previously to have an antiviral role in insects, we chose two genes for more detailed analysis: dRUVBL1 and dXPO1.

RUVBL1 had antiviral activity in *Drosophila* and mosquito cells. Depletion of dRUVBL1 in adult flies converted a non-pathogenic infection by WNV-KUN or VSV into a pathogenic infection with increased mortality and viral replication. These data suggest that RUVBL1 has a highly conserved role in antiviral defense in insects, including mosquito vectors. RUVBL1 is an AAA+ ATPase implicated in many cellular pathways [43] and that interacts with a number of other molecules that impact its function. By methodically suppressing each of its known interacting partners, we found that components of the Tip60 chromatin-remodeling complex (TIP60, EP400 and RUVBL2) that regulates transcription [43] were antiviral against multiple viruses in *Drosophila* and mosquito cells. dTip60 also was antiviral in adult flies. Furthermore, silencing of RUVBL1 led to increased viral infection in human cultured cells and primary mouse neurons. Silencing of TIP60 in human cells also rendered them more susceptible to WNV infection. Together, these results suggest a conserved role for the Tip60 complex in antiviral defense across phylogeny. WNV subgenomic replicons were used to show that the requirement for these genes in restriction is downstream of entry and upstream of viral assembly, suggesting a restriction of translation, polyprotein processing and/or RNA replication. While further investigation is required to determine the Tip60 targets that are responsible for the antiviral activity and the precise step of the lifecycle impacted, the identification of a chromatin remodeling complex as broadly antiviral is intriguing. Innate immunity is controlled, in large part, through the tight regulation of sequential gene expression programs that have effector function to restrict pathogen replication. We recently characterized a complex and rapid transcriptional antiviral host program active in insects that includes both primary responses which are translation-independent and secondary responses that are translation-dependent [31]. Half of this response was controlled at the level of transcriptional pausing, which also plays a role in innate immune responses in mammals [31], [83], [84], [85]. This antiviral transcriptional program was active against a broad panel of viruses, as we found with the Tip60 complex here. Thus, we hypothesize that the Tip60 chromatin remodeling complex may contribute to the orchestration of this sophisticated antiviral transcriptional response.

A recent study found that RUVBL2 was antiviral against influenza virus by interfering with nucleoprotein (NP) oligomerization that drives viral RNA polymerase activity in the nucleus; however, in contrast to our findings, this effect was independent of RUVBL1 function [86]. This may be a distinct and direct role for RUVBL2 in influenza replication independent of the role for the Tip60 complex in antiviral defense.

Many viruses, including those used in our studies inhibit host transcriptional responses to prevent the induction of antiviral mRNAs including IFN genes. Whether viruses target this Tip60 complex to block an antiviral transcriptional program is unknown. Tip60 is degraded by a number of nuclear viruses including HIV, adenovirus, papilloma virus and cytomegalovirus to promote viral replication [87], [88], [89], [90]. This is thought to alleviate its repression of early gene transcription. Whether these virus interactions alter the activity of the Tip60 complex on antiviral gene expression remains unknown.

The second broadly-acting VRF we investigated was XPO1. At the organismal level depletion of dXPO1 enhanced viral replication and mortality by both WNV-KUN and VSV. Data from yeast and humans suggest that XPO1 is a nuclear export receptor responsible for the translocation of RNAs and proteins from the nucleus to the cytoplasm [91], [92]. However, more recent studies have suggested that the mRNA cargo dependent on XPO1 is limited [71], [93]. Inhibition of XPO1 either by RNAi or using the specific inhibitor LMB, which blocks the nuclear export function of XPO1, resulted in increased viral infection, which suggests the antiviral role of XPO1 is at the step of nuclear export. As the vector-borne RNA viruses studied here replicate exclusively in the cytoplasm, we hypothesize that XPO1 transports cellular mRNAs critical for an antiviral response. Indeed, viruses including VSV (used in our study), HIV, VEEV, ebolavirus and picornaviruses inhibit nuclear export of antiviral genes including ISG mRNAs required for defense in mammalian cells [94], [95], [96], [97], [98], [99], [100], [101]. Consistent with this, LMB inhibition of XPO1 mediated export in human cells suppressed the export of IFNα1 mRNA [99].

While a role for nuclear export in antiviral immunity has been described in mammalian cells, its function in insect immunity was unknown. To identify the particular mRNAs responsible for the antiviral effects of XPO1 in *Drosophila* we mined a microarray study of *Drosophila* cells that found less than 2% of the mRNAs tested (85 mRNAs) exhibited nuclear export dysregulation upon LMB treatment [71]. Depletion of one XPO1-dependent mRNA, dALDOA (Aldolase A), resulted in enhanced virus infection in *Drosophila* cells. This suggests that the transport of dALDOA mRNA plays a role in the innate immune response to vector-borne viral infections. As part of the glycolysis pathway, ALDOA enzymatically cleaves fructose 1,6-bisphosphate (F-1,6-BP) into glyceraldehyde 3-phosphate (G3P) and dihydroxyacetone phosphate (DHAP). Our RNAi and pharmacological experiments suggested the mechanism of viral suppression by ALDOA was independent of its effects on glycolysis. Future studies will be required to define mechanistically how ALDOA acts to inhibit viral infections in insect cells.

Collectively, we have begun to describe a series of conserved pathways, including transcriptional pausing, chromatin remodeling and RNA export that likely regulate the expression of gene sets whose products are antiviral, perhaps in a direct way. For the most part, virus-host interaction studies have often concentrated on proteins that interact directly with the virus. Our work has revealed pathways that orchestrate larger responses, and this confers potent antiviral activity against a broad range of divergent viruses. Clearly, there is still much to be learned about the cellular factors critical for an innate immune response to vector-borne viruses in both vertebrate and invertebrate hosts. Our identification of these cell-intrinsic antiviral genes restricting WNV, and in many cases additional viruses, provides new opportunities for understanding the control mechanisms and larger antiviral programs active against globally relevant classes of emerging viral pathogens.

## Materials and Methods

### Ethics statement

This animal studies were carried out in strict accordance with the recommendations in the Guide for the Care and Use of Laboratory Animals of the National Institutes of Health. The protocol was approved by the Institutional Animal Care and Use Committee at the Washington University School of Medicine (Assurance Number: A3381-01).

### Cells, antibodies, and reagents

DL1 and Aag-2 cells were grown as previously described [31]. BHK, U2OS, and 293T cells were maintained as previously described [102]. 293T cells harboring WNV subgenomic replicon were maintained as previously described [74], [75]. Cerebellar granule cell neurons from neonatal (E6) wild-type C57BL/6 mice were generated from cerebella dissected in HBSS and dissociated in 1 mg/ml trypsin with 125 U/ml DNAse (Sigma, St. Louis, MO) for 20 min. Enzymatic digestion was quenched with DMEM/10% FCS and the tissue was pelleted, washed in HBSS, dissociated by trituration through a P-200 pipette tip and layered on a Percoll gradient. Cells were plated in neurobasal media (Gibco) supplemented with B-27 serum-free supplement (Gibco) on poly-D-lysine (PDL)-treated dishes for 1 hour to remove adherent glial cells. Nonadherent cells were then washed in HBSS, counted plated on PDL-coated wells in serum-free DMEM (supplemented with N2 growth medium (Gibco, Grand Island, NY) and 20 mM KCl. Cultures were >95% pure and were used 3 to 4 days later for lentivirus infections [103]. Antibodies were obtained from the following sources, anti-WNV NS1 (9-NS1; [37]), anti-RVFV N (1D8 – gift from C. Schmaljohn), anti-hTIP60 (abcam, ab23886) and Alexa-488 donkey anti-mouse secondary (Jackson Immunochemicals). The following inhibitors were used: Leptomycin B (SIGMA) 50 ng/ml; Dichloracetic Acid (SIGMA) 60 µM; Hexokinase II inhibitor II (Calbiochem) 0.1 mM.

### Virus stocks

West Nile virus (WNV lineage I strain 3000.0259 New York 2000) was generated in BHK cells, concentrated using Centricon Plus-70 (Millipore), and ultracentrifuged through a sucrose cushion as described previously [104]. The WNV-KUNV isolate (CH16532) was a generous gift of R. Tesh (World Reference Center of Emerging Viruses and Arboviruses, Galveston, TX) was propagated using the same protocol as WNV. DENV (gift from M. Garcia-Blanco) was grown as previously described [24]. SINV was propagated as previously described [25]. RVFV strain MP12 was propagated as described [41]. VSV was grown as described [40]. All MOIs were determined on BHK cells.

### *Drosophila* RNAi screen

For the primary WNV screen dsRNAs targeting 13,071 genes were pre-arrayed in thirty-two 384-well plates at 250 ng per well (Ambion). 16,000 DL1 cells were seeded in serum-free Schneider's media (10 uL/well). One hour later complete media was added (20 uL/well). Three days post plating, cells were infected with WNV at an MOI of 10 (10 uL/well). 48 hours post infection cells were fixed (4% formaldehyde), and a mAb against WNV NS1 (9-NS1) was used to identify infected cells (anti-mouse Alexa-fluor488 (Jackson Immunochemicals)) and counterstained with Hoechst 33342 to monitor nuclei. 3 images per well were captured at 20× using an automated microscope (ImageXpressMicro) and analyzed using MetaXpress software. Average infection and nuclei number were calculated for each site and averaged for each well. The percent infection was log-transformed, and the median and interquartile range were used to calculate a z-score: (log_10_(%infection)-log_10_(median))/(IQR*0.74) for each plate. The entire screen was performed in duplicate and those wells with Robust Z-scores≥to 2.0 or ≤to −2.0 in both replicates were considered ‘hits’.

Similar to the primary screen, secondary screen plates were arrayed with dsRNA (250 ng) targeting a different region of the genes identified in the primary screen (DRSC). WNV infections were performed in duplicate at a higher (20) and lower (5) MOI (18% and 4% respectively). Infections with other viruses used the same protocol as WNV and were fixed at the following hours post-infection: WNV-KUN - 48 hrs (MOI = 10), DENV - 72 hrs (MOI = 10), SINV – 40 hrs (MOI = 5), VSV – 24 hrs (MOI = 1), RVFV MP12 – 30 hrs (MOI = 1). Robust Z-scores in each duplicate viral infection set of ≥1.5 or ≤1.5 in duplicate (∼40% change) were considered ‘hits’ (p<0.009); none of the negative controls (non-targeting) were identified as positive, and all of the positive controls (dsRNA against virus genome) were identified.

### Bioinfomatic analysis

The functional annotation and clustering of WNV ‘hits’ was performed using the DAVID Bioinfomatics resource. Homologene (NCBI) was used to identify orthologs. Gene Cluster and TreeView were used to generate heat maps.

### Adult infections

All flies were maintained on standard medium at room temperature. Flies carrying UAS-dXPO1 IR (VDRC v3347) or UAS-dRUVBL1 IR (VDRC v105408) were crossed to heat shock (HS)-GAL4 flies (Bloomington) at room temperature. On the day of injection, the progeny were heat-shocked at 37°C for 1 hour and shocked every 2 days throughout the experiment. Adults of the stated genotypes were challenged with WNV-KUN or VSV as previously described [22]. Groups of at least 20 flies were challenged for mortality studies. For viral titers, groups of 15 flies per experimental treatment were crushed and processed at 6 days post-infection for plaque assays on BHK cells [31].

### Quantitative PCR

Total RNA was isolated from infected cells using Trizol (Invitrogen). For northern blots, RNA species were analyzed as previously described [105]. For RT-qPCR, cDNA was generated using random hexamers to prime reverse transcription reactions using MMLV reverse transcriptase. cDNA samples were treated with 100 U DNase I (Qiagen) according to manufacturer's protocol. Quantitative PCR (qPCR) was performed with the cDNA using Power SYBR Green PCR Master Mix (Applied Biosystems) and primers targeting VSV and WNV (VSV For-CGGAGGATTGACGACTAATGC, Rev-ACCATCCGAGCCATTCGA: WNV For-ACATCAAACGTGGTTGTTCCGCTG, Rev-TTGAGGCTAGAGCCAAGCATAGCA) in accordance with manufacturer's protocol. qPCR conditions were as follows; initial 94°C for 5 min, then 30 cycles of 94°C for 30 sec, 55°C for 30 sec, and 72°C for 30 sec. Relative viral copy numbers were generated by normalizing to cells treated with control dsRNA.

### Mammalian RNAi

For siRNA treatments, mammalian cells were reverse transfected with 20 nM siRNA (Ambion: Negative Control #1 (AM4611), GFP (AM4626), XPO1 (s14937), RUVBL1 (s16370)), KAT5 (s20630, s20631) using HiPerfect according to the manufacturers protocol (Qiagen). 60 hours post-transfection, for immunofluorescence, cells were replated in a 96-well format and infected with either VSV or WNV-KUN virus 12 hours later. For FACS the cells were infected with WNV for 24 hours and stained for NS1 or TIP60 [82] or infected and processed for northern blot or immunoblot at 12 hr p.i. for VSV and 20 hr p.i. for WNV-KUN.

For shRNA treatments, lentiviruses (pLK0.1) encoding shRNA targeting RUVBL1 (clone 1: GCTGGAGATGTGATTTACATT; clone 2: GCTGGCAAAGATCAATGGCAA; clone 3: GCCACAGAGTTTGACCTTGAA; clone 4: GCAAGATATTCTGTCTATGAT) or a control (luciferase) were obtained from RNAi Core facility at Washington University School of Medicine. Lentivirus particles were generated after co-transfection of HEK-293T cells with packaging plasmids. Supernatants were collected at 48 hours later and added to neuron cultures. Three days after transduction, neurons were infected with WNV (New York 1999 strain) at an MOI of 0.1. One day later, supernatants were harvested and titered for virus infection by focus-forming assay [106].

### Drug treatments

One day prior to infection U2OS or DL1 cells were seeded in 96-well plates at 20,000 or 70,000 cells per well respectively. Leptomycin B (SIGMA) was added at 50 ng/ml to mammalian cells cells 2 hr prior to infection with WNV-KUN or VSV. Dichloroacetic Acid (SIGMA) or Hexokinase II inhibitor II (Calbiochem) were added to U2OS or DL1 cells respectively, 30 minutes prior to infection. Cells were fixed 20 (WNV-KUN) or 12 (VSV) hr post infection, stained and imaged as previously described.

## Supporting Information

Table S1 **Complexes identified in the screen.** Full list of complexes identified along with the genes identified in the primary screen and those validated in the secondary screens.
(PDF)

Table S2 **Genes identified and validated in the genome-wide RNAi screen against WNV.** Full list of genes identified in the genome-wide screen and validated in secondary screens with the average Robust Z scores shown.
(PDF)

Table S3 **Genes having antiviral activity against WNV were tested against a panel of arboviruses.** Robust Z scores for primary and secondary screens shown. Genes that were not tested are left blank and those in bold were validated in all screens.
(PDF)

Table S4 **List of VRFs identified in other screens.** A compilation of genes identified in published screens with activity against flaviviruses are shown with those that have orthologs in Drosophila listed in red. Little overlap is observed.
(PDF)

Text S1 **Compilation of seven supplemental figures, their legends and associated methods are shown.**
(PDF)
