# Supplementary material for: Genome-Wide RNAi Screen Identifies Broadly-Acting Host Factors That Inhibit Arbovirus Infection
Source: PLoS Pathog. 2014 Feb 13;10(2):e1003914. doi: 10.1371/journal.ppat.1003914 (PMC3923753; doi:10.1371/journal.ppat.1003914)
Supplement: Table S1 — Complexes identified in the screen. Full list of complexes identified along with the genes identified in the primary screen and those validated in the secondary screens. (PDF) [file ppat.1003914.s001.pdf]

Table S1. Complexes identified in the screen.

Complex / Process	Genes primary screen	Genes validated
Vacuolar-type H+-ATPase	Vha100-1, Vha100-2, **Vha55**, VhaPPA1-1, **VhaAC39**, Vha13, Vha14, Vha26, **VhaSFD, VhaAC45, VhaM8.9**	VhaAC45, VhaSFD, VhaM8.9, Vha55, VhaAC39
Signal Recognition complex/ Signal peptidase complex	**Srp19, Srp54K,** Srp9, Srp14, **SrpBeta, Srp72, Spase25,** Spase22-23	SrpBeta, Srp72, Srp19, Srp54K, Spase25
Translation initiation	**eIF-S9, eIF5B, eIF2B-beta**, eIF2B-epsilon, **eIF4G, eIF2B-gamma, eIF-4B, CG17737**	eIF2B-gamma, eIF-4B, CG17737, eIF2B-Beta, eIF3-S9, eIF4G, eIF5B
Mediator complex	**MED4, MED7, MED8,** MED10, MED14, MED17, MED22	MED8, MED4, MED7
Arp2/3 complex	Arpc3B, Arp66B, **Arp14D**	Arp14D
Tafs	**Taf6, Taf5**, Taf12	Taf5, Taf6
