# Supplementary material for: Genome-Wide RNAi Screen Identifies Broadly-Acting Host Factors That Inhibit Arbovirus Infection
Source: PLoS Pathog. 2014 Feb 13;10(2):e1003914. doi: 10.1371/journal.ppat.1003914 (PMC3923753; doi:10.1371/journal.ppat.1003914)
Supplement: Table S2 — Genes identified and validated in the genome-wide RNAi screen against WNV. Full list of genes identified in the genome-wide screen and validated in secondary screens with the average Robust Z scores shown. (PDF) [file ppat.1003914.s002.pdf]

Table S2. Genes identified and validated in the genome-wide RNAi screen against WNV.

gene	CG ID	Human Homolog	Mosquito Ortholog	Primary Avg z-score inf	WNV secondary Avg z-score inf
CG17737	CG17737	EIF1	AgaP_AGAP006459	3.15	4.57
Taf6	CG32211	TAF6	AgaP_AGAP004254	3.08	2.94
CG32113	CG32113	VPS13D	AgaP_AGAP009780	2.98	2.34
skd	CG9936	MED13L	AgaP_AGAP006436	2.86	3.07
yellow-c | l(2)35Bg	CG4182		AgaP_AGAP010280	2.85	2.18
CrebA	CG7450		AgaP_AGAP011038	2.84	1.37
skpA	CG16983	LOC100506332	AgaP_AGAP008719	2.74	1.44
CG33123	CG33123	LARS	AgaP_AGAP008297	2.73	2.77
Aats-tyr	CG4561	YARS	AgaP_AGAP003003	2.72	1.88
CG1789	CG1789	UTP11L	AgaP_AGAP004336	2.71	3.11
pont	CG4003	RUVBL1	ENSANGG00000022263	2.62	2.53
CG14516	CG14516		AgaP_ENSANGG00000018759	2.60	2.49
PPP4R2r	CG2890	PPP4R2	ENSANGG00000019508	2.55	2.41
CG3224	CG3224	ZNF593	AgaP_AGAP003698	2.52	1.76
CG7394	CG7394	DNAJC19	AgaP_AGAP003533	2.51	2.14
CG17119	CG17119	CTNS	AgaP_AGAP004115	2.50	2.86
CG9053	CG9053	TMED5	AgaP_AGAP001505	2.49	3.02
MED8	CG13867	MED8	AgaP_AGAP005909	2.49	2.23
CG3876	CG3876	FRAG1	AgaP_AGAP007869	2.45	1.67
CG10158	CG10158	FGFR1OP2	AgaP_AGAP012118	2.40	2.81
phl	CG2845	BRAF	AgaP_AGAP004699	2.38	2.27
CG6495	CG6495		AgaP_AGAP007887	2.37	1.97
Rab-RP3	CG7062	RAB43	AgaP_AGAP007096	2.35	2.83
cn	CG1555	KMO	KMO_ANOGA	2.35	1.71
CG3335	CG3335	RBM19	AgaP_AGAP005249	2.34	3.13
CG11526	CG11526	FAM40A	AgaP_AGAP007902	2.32	4.07
CG10646	CG10646	TSSC1	AgaP_AGAP006239	2.32	2.09
CG5326	CG5326		AgaP_AGAP011812	2.31	3.56
Taf5	CG7704	TAF5	AgaP_AGAP011678	2.30	3.38
CG6094	CG6094	ICT1	AgaP_AGAP008425	2.29	2.76
Axn	CG7926	AXIN2	AgaP_AGAP002123	2.24	3.12
Spt20	CG17689		AgaP_AGAP012403	2.23	2.64
emb	CG13387	XPO1	AgaP_AGAP009929	2.17	2.90
CG11920	CG11920	IMP4	AgaP_AGAP003890	2.15	3.23
l(2)k07824	CG7989	UTP18	AgaP_AGAP009046	2.14	3.73
CG11837	CG11837	DIMT1L	AgaP_AGAP004465	2.14	1.58
cnk	CG6556		AgaP_AGAP009167	2.12	2.25
CG7115	CG7115	PPM1L	ENSANGG00000018281	2.08	3.23
eIF2B-gamma	CG8190	EIF2B3	AgaP_AGAP005210	2.06	2.03
**eIF3-S9**	**CG4878**	**EIF3B**	**EIF3B_ANOGA**	**2.27**	**Fig S1F**
**eIF5B**	**CG10840**	**EIF5B**	**AgaP_AGAP004824**	**2.47**	**Fig S1F**
**eIF2B-beta**	**CG2677**	**EIF2B2**	**AgaP_AGAP007097**	**2.67**	**Fig S1F**
**eIF2B-epsilon**	**CG3806**	**EIF2B5**	**ENSANGG00000017368**	**2.75**	
**MED4**	**CG8609**	**MED4**	**AgaP_AGAP004063**	**2.65**	**Fig S1F**

**MED7**	**CG31390**	**MED7**	**AgaP_AGAP002823**	**2.50**	**Fig S1F**
**MED10**	**CG5057**	**MED10**	**AgaP_AGAP006248**	**2.09**	
**MED14**	**CG12031**	**MED14**	**AgaP_AGAP005700**	**3.11**	
**MED17**	**CG7957**	**MED17**	**MED17_ANOGA**	**2.49**	
**MED22**	**CG3034**	**MED22**	**AgaP_AGAP004191**	**2.31**	
**Arpc3B**	**CG8936**	**ARPC3**	**AgaP_AGAP001712**	**2.52**	
U26	CG13401	AASDH	AgaP_AGAP010071	-2.16	-1.66
maf-S	CG9954	MAFK	AgaP_AGAP010405	-2.17	-1.41
IntS12	CG5491	INTS12	AgaP_AGAP004359	-2.18	-3.94
Trn-SR	CG2848	TNPO3	AgaP_AGAP003576	-2.19	-2.08
Rab7	CG5915	RAB7A	AgaP_AGAP001617	-2.22	-1.36
CG6051	CG6051		AgaP_AGAP003678	-2.22	-1.51
HdacX	CG31119	HDAC11	AgaP_AGAP001736	-2.30	-4.47
PGRP-SC1a/b	CG14746	PGLYRP1	PGRPS3	-2.32	-1.79
br	CG11491		ENSANGG00000016940	-2.33	-2.59
Cht9	CG10531		AgaP_AGAP004876	-2.34	-2.14
CG11455	CG11455		AgaP_AGAP009824	-2.37	-3.24
CG8237	CG8237	FAM8A1	AgaP_AGAP011364	-2.37	-3.75
chic	CG9553		AgaP_AGAP009861	-2.41	-1.90
Ugt35a	CG6644	UGT1A4		-2.44	-3.75
CG9175	CG9175	PREB	AgaP_AGAP009942	-2.52	-3.59
NP15.6	CG6008		ENSANGG00000015418	-2.63	-3.27
CG7219	Spn28D		SRPN4/5/6/16	-2.64	-2.82
CG9311	CG9311	PTPN23	AgaP_AGAP011586	-2.67	-2.00
spen	CG18497	SPEN		-2.68	-4.44
VhaM8.9	CG8444	ATP6AP2	AgaP_AGAP003430	-2.69	-1.45
Bsg	CG31605		AgaP_AGAP008408	-2.80	-2.94
PICK1	CG6167	PICK1	AgaP_AGAP007832	-2.83	-3.21
Kap-alpha3	CG9423	KPNA4	AgaP_AGAP001273	-2.87	-2.89
cos	CG1708	KIF7	AgaP_ENSANGG0000016509	-2.92	-1.47
CG2145	CG2145	P11	AgaP_AGAP002925	-2.96	-2.11
CG5284	CG5284	CLCN3	AgaP_AGAP005777	-3.06	-2.87
CG9911	CG9911	TXNDC4	AgaP_AGAP000909	-3.08	-2.52
CG31523	CG31523	ELOVL7	AgaP_AGAP004372	-3.18	-1.48
l(1)G0155	CG1515	YKT6	AgaP_AGAP000392	-3.20	-3.08
Herp	CG14536	HERPUD2		-3.21	-4.24
Tango5	CG32675	TMEM49	AgaP_AGAP010304	-3.21	-3.01
CG17766	CG17766	WDR7	AgaP_AGAP008003	-3.27	-2.65
CG3036	CG3036		AgaP_AGAP009498	-3.36	-4.19
CG32276	CG32276	SERP2	AgaP_AGAP001548	-3.38	-2.05
CG11820	CG11820		AgaP_AGAP011757	-3.38	-4.14
CG5021	CG5021	FAM18A	AgaP_AGAP012432	-3.41	-7.26
CG6995	CG6995	SLTM	AgaP_AGAP001298	-3.50	-2.62
Or94a	CG17241		GPROR34	-3.50	-7.74
CG6488	CG6488	COG8	AgaP_AGAP011488	-3.51	-2.82
Rab5	CG3664	RAB5C	AgaP_AGAP007901	-3.54	-4.75
CG5745	CG5745	TBC1D22B	AgaP_AGAP004522	-3.81	-1.64
CG7816	CG7816	SLC39A13	AgaP_AGAP003300	-3.84	-1.75
CG11134	CG11134	PTPN23	AgaP_AGAP011586	-3.86	-2.79
Chc	CG9012	CLTC	AgaP_AGAP003021	-4.01	-2.63

vib	CG5269	PITPNA	AgaP_AGAP001957	-4.08	-2.47
Vps16A	CG8454	VPS16	AgaP_AGAP000529	-4.10	-3.35
CG7456	CG7456	COG4	AgaP_AGAP008875	-4.11	-5.35
Rack1	CG7111	GNB2L1	AgaP_AGAP010173	-4.16	-3.66
VhaSFD	CG17332	ATP6V1H	AgaP_AGAP009486	-4.22	-3.41
alpha-Est9	CG1128		AgaP_AGAP006227	-4.30	-5.34
lace	CG4162	SPTLC2	AgaP_AGAP007941	-4.40	-4.36
SNF1A	CG3051	PRKAA2	AgaP_AGAP002686	-4.42	-5.43
CG8176	CG8176	FCHO2	AgaP_AGAP002024	-4.42	-1.72
CG17328	CG17328		AgaP_AGAP009130	-4.45	-1.62
wmd	CG3957	STRAP	AgaP_AGAP005029	-4.56	-3.40
lwr	CG3018	UBE2I	AgaP_AGAP011076	-4.76	-3.58
Arp14D	CG9901	ACTR2	AgaP_AGAP000985	-4.76	-5.48
CG9773	CG9773	UNC50	AgaP_AGAP002901	-4.78	-7.25
CG3149	CG3149	RFT1	AgaP_AGAP004419	-5.04	-2.85
CG11963	CG11963	SUCLA2	AgaP_AGAP004744	-5.16	-3.66
CG4557	CG4557	TMF1	AgaP_AGAP000660	-5.30	-2.19
Bap	CG12532	AP1B1		-5.34	-3.55
Slh	CG3539	SCFD1	AgaP_AGAP011358	-5.47	-1.56
CG8029	CG8029		AgaP_AGAP003879	-5.48	-5.15
AP-50	CG7057	AP2M1	AgaP_AGAP007131	-5.58	-3.64
Syx5	CG4214	STX5	AgaP_AGAP008756	-5.60	-2.40
CG1868	CG1868		AgaP_AGAP008973	-5.63	-3.37
CG4645	CG4645	YIPF1	AgaP_AGAP006951	-5.68	-3.17
Spase25	CG1751	SPCS2	AgaP_AGAP011842	-5.82	-3.97
sec23	CG1250	SEC23A	AgaP_AGAP001759	-5.82	-2.20
Aats-arg	CG9020	RARS	AgaP_AGAP004708-PA	-6.09	-1.60
SrpRbeta	CG33162	SRPRB	AgaP_AGAP006688	-6.19	-5.33
shi	CG18102	DNM1	AgaP_AGAP003018	-6.26	-2.50
Vha26	CG1088	ATP6V1E1	AgaP_AGAP002401	-6.91	-2.25
Syx7	CG5081	STX7	AgaP_AGAP005543	-7.27	-4.02
CG40127	CG40127		AgaP_AGAP006389	-7.27	-7.84
CG2926	CG2926		AgaP_AGAP010697	-8.48	-2.19
Srp72	CG5434	SRP72	AgaP_AGAP001939	-8.75	-5.21
eIF-4E	CG4035		AgaP_AGAP007172	-9.63	-4.32
mr	CG3060	ANAPC2	AgaP_AGAP005048	-9.64	-8.28
Gtp-bp	CG2522	SRPR	AgaP_AGAP010894	-9.87	-4.01
CG4484	CG4484		AgaP_AGAP010854	-10.26	-2.50
**Vha100-1**	**CG1709**	**ATP6V0A1**	**AgaP_AGAP003711**	**-3.33**	
**Vha100-2**	**CG18617**	**ATP6V0A4**	**AgaP_AGAP001587**	**-4.50**	
**Vha55**	**CG17369**	**ATP6V1B2**	**AgaP_AGAP002884**	**-4.11**	**Fig S1F**
**VhaPPA1-1**	**CG7007**	**ATP6V0B**	**AgaP_AGAP009334**	**-9.84**	
**VhaAC39**	**CG2934**	**ATP6V0D1**	**AgaP_AGAP000721**	**-5.51**	**Fig S1F**
**Vha13**	**CG6213**	**ATP6V1G2**	**AgaP_AGAP001823**	**-5.02**	
**Vha14**	**CG8210**	**ATP6V1F**	**AgaP_AGAP002473**	**-6.63**	
**Srp19**	**CG4457**	**SRP19**	**AgaP_AGAP006895**	**-5.46**	**Fig S1F**
**Srp54K**	**CG4659**	**SRP54**	**AgaP_AGAP004610**	**-12.55**	**Fig S1F**
**Srp9**	**CG8268**		**AgaP_AGAP007492**	**-4.80**	
**Srp14**	**CG5417**		**AgaP_AGAP008339**	**-6.27**	
**Arp66B**	**CG7558**	**ACTR3**	**AgaP_AGAP005110**	**-4.48**	
**eIF4G**	**CG10811**	**EIF4G1**	**AgaP_AGAP002502**	**-3.18**	**Fig S1F**
