# Supplementary material for: Genome-Wide RNAi Screen Identifies Broadly-Acting Host Factors That Inhibit Arbovirus Infection
Source: PLoS Pathog. 2014 Feb 13;10(2):e1003914. doi: 10.1371/journal.ppat.1003914 (PMC3923753; doi:10.1371/journal.ppat.1003914)
Supplement: Table S3 — Genes having antiviral activity against WNV were tested against a panel of arboviruses. Robust Z scores for primary and secondary screens shown. Genes that were not tested are left blank and those in bold were validated in all screens. (PDF) [file ppat.1003914.s003.pdf]

Table S3. Genes that are antiviral against WNV were tested against a panel of arboviruses.

gene	CG ID	Human Homolog	Mosquito Ortholog	Primary Avg z-score inf	WNV secondary Avg z-score inf	KUN Avg z-score inf	DEN Avg z-score inf	SIN Avg z-score inf	RVFV Avg z-score inf	VSV Avg z-score inf
Aats-arg	CG9020	RARS	AgaP_AGAP004708-PA	-6.09	-1.60			4.54	-2.23	-0.83
**Aats-tyr**	**CG4561**	**YARS**	**AgaP_AGAP003003**	**2.72**	**1.88**	**1.85**	**2.19**	**1.33**	**1.78**	**1.85**
alpha-Est9	CG1128		AgaP_AGAP006227	-4.30	-5.34			1.41	-0.26	
AP-50	CG7057	AP2M1	AgaP_AGAP007131	-5.58	-3.64			-3.09	-2.80	2.25
Arp14D	CG9901	ACTR2	AgaP_AGAP000985	-4.76	-5.48	0.68	-3.62	0.38	-2.21	
Axn	CG7926	AXIN2	AgaP_AGAP002123	2.24	3.12	1.20	-1.23	1.40	-0.06	-0.69
Bap	CG12532	AP1B1		-5.34	-3.55			-1.97	-2.68	1.96
br	CG11491		ENSANGG00000016940	-2.33	-2.59	-0.25	-0.16	1.26	-0.55	0.53
Bsg	CG31605		AgaP_AGAP008408	-2.80	-2.94	1.94	1.51	0.60	-0.21	1.60
CG10158	CG10158	FGFR1OP2	AgaP_AGAP012118	2.40	2.81	2.11	1.50	0.62	1.51	
CG10646	CG10646	TSSC1	AgaP_AGAP006239	2.32	2.09	0.10	1.44	2.07	-0.08	0.90
CG11134	CG11134	PTPN23	AgaP_AGAP011586	-3.86	-2.79	-0.69	0.02	-2.38	0.36	-0.36
CG11455	CG11455		AgaP_AGAP009824	-2.37	-3.24	0.68	0.71	0.02	0.69	1.99
CG11526	CG11526	FAM40A	AgaP_AGAP007902	2.32	4.07	4.13	2.42	0.74	0.35	-1.15
CG11820	CG11820		AgaP_AGAP011757	-3.38	-4.14	0.64	1.23	0.58	-0.03	-1.16
CG11837	CG11837	DIMT1L	AgaP_AGAP004465	2.14	1.58	-0.08	-0.01	-0.36	-0.33	0.38
CG11920	CG11920	IMP4	AgaP_AGAP003890	2.15	3.23	2.34	0.39	2.00	0.95	0.52
CG11963	CG11963	SUCLA2	AgaP_AGAP004744	-5.16	-3.66	0.64	0.49	-2.54	0.04	0.54
CG14516	CG14516		AgaP_ENSANGG00000018759	2.60	2.49	0.51	0.15	1.70	0.01	-0.57
**CG17119**	**CG17119**	**CTNS**	**AgaP_AGAP004115**	**2.50**	**2.86**	**2.87**	**2.99**	**1.38**	**1.96**	**3.84**
CG17328	CG17328		AgaP_AGAP009130	-4.45	-1.62	-0.05	0.36	0.78	-0.28	-0.51
**CG17737**	**CG17737**	**EIF1**	**AgaP_AGAP006459**	**3.15**	**4.57**	**3.46**	**2.93**	**2.46**	**1.79**	**2.27**
CG17766	CG17766	WDR7	AgaP_AGAP008003	-3.27	-2.65			-2.97	-2.42	-0.36
CG1789	CG1789	UTP11L	AgaP_AGAP004336	2.71	3.11	1.59	0.11	3.07	0.73	-1.58
CG1868	CG1868		AgaP_AGAP008973	-5.63	-3.37	0.93	-1.82	-0.77	-0.33	-0.48
CG2145	CG2145	P11	AgaP_AGAP002925	-2.96	-2.11	0.36	0.50	1.52	0.02	-0.11
CG2926	CG2926		AgaP_AGAP010697	-8.48	-2.19			-8.06	0.51	2.64
CG3036	CG3036		AgaP_AGAP009498	-3.36	-4.19	0.57	-2.01	1.78	0.13	-0.01
CG3149	CG3149	RFT1	AgaP_AGAP004419	-5.04	-2.85	0.11	1.50	-0.80	-0.43	-0.18
CG31523	CG31523	ELOVL7	AgaP_AGAP004372	-3.18	-1.48	-0.07	0.81	-1.21	-0.27	0.38
CG32113	CG32113	VPS13D	AgaP_AGAP009780	2.98	2.34	2.05	-0.75	1.33	-0.09	0.53
CG3224	CG3224	ZNF593	AgaP_AGAP003698	2.52	1.76	2.03	-0.02	2.36	2.19	2.55
CG32276	CG32276	SERP2	AgaP_AGAP001548	-3.38	-2.05	-0.98	0.43	0.94	-0.54	0.19
CG33123	CG33123	LARS	AgaP_AGAP008297	2.73	2.77	2.34	-0.44	1.73	-0.48	0.71
CG3335	CG3335	RBM19	AgaP_AGAP005249	2.34	3.13	2.15	0.70	2.47	1.29	0.30
CG3876	CG3876	FRAG1	AgaP_AGAP007869	2.45	1.67	-0.62	1.39	-0.49	0.09	0.09
CG40127	CG40127		AgaP_AGAP006389	-7.27	-7.84	-1.01	-4.40	-2.28	-2.89	
CG4484	CG4484		AgaP_AGAP010854	-10.26	-2.50	0.80	-2.08	1.23	-0.16	0.35
CG4557	CG4557	TMF1	AgaP_AGAP000660	-5.30	-2.19			-2.19	-1.15	0.77
CG4645	CG4645	YIPF1	AgaP_AGAP006951	-5.68	-3.17	-0.68	-0.73	0.69	0.01	0.74
CG5021	CG5021	FAM18A	AgaP_AGAP012432	-3.41	-7.26	0.01	-1.07	-0.32	-0.12	-0.47
CG5284	CG5284	CLCN3	AgaP_AGAP005777	-3.06	-2.87			-9.84	-4.97	-2.82
CG5326	CG5326		AgaP_AGAP011812	2.31	3.56	1.70	0.69	0.12	0.23	-1.02
CG5745	CG5745	TBC1D22B	AgaP_AGAP004522	-3.81	-1.64	0.06	0.87	0.83	0.17	-0.12
CG6051	CG6051		AgaP_AGAP003678	-2.22	-1.51	0.47	0.57	-0.01	-0.21	
**CG6094**	**CG6094**	**ICT1**	**AgaP_AGAP008425**	**2.29**	**2.76**	**2.87**	**1.89**	**1.33**	**2.06**	**2.56**
CG6488	CG6488	COG8	AgaP_AGAP011488	-3.51	-2.82	-0.63	1.20	-0.98	0.55	0.54
CG6495	CG6495		AgaP_AGAP007887	2.37	1.97	-0.02	-0.73	0.88	0.58	-0.26
CG6995	CG6995	SLTM	AgaP_AGAP001298	-3.50	-2.62			-5.82	2.66	3.00
**CG7115**	**CG7115**	**PPM1L**	**ENSANGG00000018281**	**2.08**	**3.23**	**2.37**	**1.40**	**1.88**	**1.63**	**2.30**
CG7219	Spn28D		SRPN4/5/6/16	-2.64	-2.82	0.25	-0.63	-0.77	-1.14	0.48
CG7394	CG7394	DNAJC19	AgaP_AGAP003533	2.51	2.14	2.99	-0.92	1.75	1.65	
CG7456	CG7456	COG4	AgaP_AGAP008875	-4.11	-5.35	0.09	-0.30	-2.23	2.23	0.34
CG7816	CG7816	SLC39A13	AgaP_AGAP003300	-3.84	-1.75	0.45	0.62	1.38	0.35	0.52
CG8029	CG8029		AgaP_AGAP003879	-5.48	-5.15	-1.55	-5.41	-2.22	-2.78	-4.67
CG8176	CG8176	FCHO2	AgaP_AGAP002024	-4.42	-1.72	-0.24	-2.32	-0.64	-0.70	-0.60
CG8237	CG8237	FAM8A1	AgaP_AGAP011364	-2.37	-3.75	-0.34	0.42	0.14	-0.46	-0.53
VhaM8.9	CG8444	ATP6AP2	AgaP_AGAP003430	-2.69	-1.45			-2.83	-2.07	1.11
CG9053	CG9053	TMED5	AgaP_AGAP001505	2.49	3.02	2.43	2.32	2.33	0.94	1.40
CG9175	CG9175	PREB	AgaP_AGAP009942	-2.52	-3.59	0.24	0.77	0.40	1.19	-0.98
CG9311	CG9311	PTPN23	AgaP_AGAP011586	-2.67	-2.00			-1.40	-0.83	1.01
CG9773	CG9773	UNC50	AgaP_AGAP002901	-4.78	-7.25	-0.81	-0.14	-3.54	0.98	1.13
CG9911	CG9911	TXNDC4	AgaP_AGAP000909	-3.08	-2.52	-0.53	-1.83	-0.28	-0.12	-2.48
Chc	CG9012	CLTC	AgaP_AGAP003021	-4.01	-2.63			-5.32	-1.72	1.80
chic	CG9553		AgaP_AGAP009861	-2.41	-1.90	0.13	-0.84	1.00	-0.26	0.64
Cht9	CG10531		AgaP_AGAP004876	-2.34	-2.14	-0.79	0.98	-2.12	-0.01	0.14
cn	CG1555	KMO	KMO_ANOGA	2.35	1.71			4.21	-1.17	1.17
cnk	CG6556		AgaP_AGAP009167	2.12	2.25	2.13	1.40	0.41	-1.17	-2.77

cos	CG1708	KIF7	AgaP_ENSANGG00000016509	-2.92	-1.47	0.46	-1.72	1.19	-0.72	
CrebA	CG7450		AgaP_AGAP011038	2.84	1.37	0.12	-0.97	-0.17	-0.49	-0.09
eIF-4E	CG4035		AgaP_AGAP007172	-9.63	-4.32			-1.52	-2.68	-2.18
eIF2B-gamma	CG8190	EIF2B3	AgaP_AGAP005210	2.06	2.03			5.31	3.10	2.44
**emb**	**CG13387**	**XPO1**	**AgaP_AGAP009929**	**2.17**	**2.90**	**3.14**	**3.16**	**2.68**	**2.39**	**3.73**
Gtp-bp	CG2522	SRPR	AgaP_AGAP010894	-9.87	-4.01	-0.75	-5.97	-1.53	0.51	-2.17
HdacX	CG31119	HDAC11	AgaP_AGAP001736	-2.30	-4.47	-0.54	0.42	0.19	-0.42	-1.88
Herp	CG14536	HERPUD2		-3.21	-4.24	-0.03	-1.69	1.53	-0.01	-0.41
IntS12	CG5491	INTS12	AgaP_AGAP004359	-2.18	-3.94	0.06	-0.75	-0.70	-0.29	0.10
Kap-alpha3	CG9423	KPNA4	AgaP_AGAP001273	-2.87	-2.89	0.02	-2.21	-0.59	0.21	
l(1)G0155	CG1515	YKT6	AgaP_AGAP000392	-3.20	-3.08	-0.68	-0.58	-0.64	-0.73	0.67
l(2)k07824	CG7989	UTP18	AgaP_AGAP009046	2.14	3.73	1.86	1.43	2.63	1.34	0.51
lace	CG4162	SPTLC2	AgaP_AGAP007941	-4.40	-4.36			-7.23	-0.78	1.47
lwr	CG3018	UBE2I	AgaP_AGAP011076	-4.76	-3.58	0.20	-0.42	0.84	0.56	
maf-S	CG9954	MAFK	AgaP_AGAP010405	-2.17	-1.41	-0.90	-0.38	-0.32	-0.69	-0.70
MED8	CG13867	MED8	AgaP_AGAP005909	2.49	2.23	1.70	1.74	3.25	1.46	0.66
mr	CG3060	ANAPC2	AgaP_AGAP005048	-9.64	-8.28	0.08	1.45	0.31	0.83	0.79
NP15.6	CG6008		ENSANGG00000015418	-2.63	-3.27	0.81	0.07	0.68	1.29	2.00
Or94a	CG17241		GPROR34	-3.50	-7.74	-0.77	0.29	-0.44	0.01	-0.01
PGRP-SC1a/b	CG14746	PGLYRP1	PGRPS3	-2.32	-1.79	0.26	-0.16	0.98	-0.43	0.20
phl	CG2845	BRAF	AgaP_AGAP004699	2.38	2.27	1.77	1.41	-0.53	0.62	1.29
PICK1	CG6167	PICK1	AgaP_AGAP007832	-2.83	-3.21	1.23	0.49	1.35	-0.88	-0.01
**pont**	**CG4003**	**RUVBL1**	**ENSANGG00000022263**	**2.62**	**2.53**	**2.55**	**3.21**	**2.91**	**2.21**	**2.96**
PPP4R2r	CG2890	PPP4R2	ENSANGG00000019508	2.55	2.41	2.53	2.77	1.55	1.61	1.25
Rab-RP3	CG7062	RAB43	AgaP_AGAP007096	2.35	2.83	0.16	0.46	0.10	0.22	-0.43
Rab5	CG3664	RAB5C	AgaP_AGAP007901	-3.54	-4.75			-9.26	-3.67	0.12
Rab7	CG5915	RAB7A	AgaP_AGAP001617	-2.22	-1.36			-3.60	-2.16	0.93
Rack1	CG7111	GNB2L1	AgaP_AGAP010173	-4.16	-3.66	-0.14	-0.86	1.10	-0.42	0.18
sec23	CG1250	SEC23A	AgaP_AGAP001759	-5.82	-2.20			-3.68	1.12	2.56
shi	CG18102	DNM1	AgaP_AGAP003018	-6.26	-2.50			-2.57	-1.49	0.97
skd	CG9936	MED13L	AgaP_AGAP006436	2.86	3.07	3.16	0.55	1.50	0.59	
skpA	CG16983	LOC10050633	AgaP_AGAP008719	2.74	1.44			-0.24	-0.12	-0.13
Slh	CG3539	SCFD1	AgaP_AGAP011358	-5.47	-1.56			-6.95	2.46	1.37
SNF1A	CG3051	PRKAA2	AgaP_AGAP002686	-4.42	-5.43			-8.04	-0.50	-2.62
Spase25	CG1751	SPCS2	AgaP_AGAP011842	-5.82	-3.97	-0.26	-6.63	1.11	-0.26	0.95
spen	CG18497	SPEN		-2.68	-4.44	-0.76	0.50	0.54	-0.57	0.31
Spt20	CG17689		AgaP_AGAP012403	2.23	2.64	2.80	0.03	0.84	0.24	0.23
Srp72	CG5434	SRP72	AgaP_AGAP001939	-8.75	-5.21	-2.59	-5.64	-2.39	0.66	-1.93
SrpRbeta	CG33162	SRPRB	AgaP_AGAP006688	-6.19	-5.33	-1.42	-4.02	0.24	1.00	-1.17
Syx5	CG4214	STX5	AgaP_AGAP008756	-5.60	-2.40			-4.95	0.81	0.81
Syx7	CG5081	STX7	AgaP_AGAP005543	-7.27	-4.02	-0.77	-1.86	-5.03	-4.64	-0.67
Taf5	CG7704	TAF5	AgaP_AGAP011678	2.30	3.38	3.08	2.52	2.55	1.04	0.97
Taf6	CG32211	TAF6	AgaP_AGAP004254	3.08	2.94	2.63	2.04	2.41	0.63	0.76
Tango5	CG32675	TMEM49	AgaP_AGAP010304	-3.21	-3.01	-0.26	-6.41	-2.28	0.69	0.69
Trn-SR	CG2848	TNPO3	AgaP_AGAP003576	-2.19	-2.08	-0.48	0.35	0.98	-0.01	0.57
U26	CG13401	AASDH	AgaP_AGAP010071	-2.16	-1.66	-0.49	-0.22	-0.32	0.06	-4.18
Ugt35a	CG6644	UGT1A4		-2.44	-3.75	-0.02	-1.36	1.28	-0.69	-0.34
Vha26	CG1088	ATP6V1E1	AgaP_AGAP002401	-6.91	-2.25			-1.56	-0.09	-0.17
VhaSFD	CG17332	ATP6V1H	AgaP_AGAP009486	-4.22	-3.41			-4.27	-1.93	-0.36
vib	CG5269	PITPNA	AgaP_AGAP001957	-4.08	-2.47			-4.98	-1.76	-0.81
Vps16A	CG8454	VPS16	AgaP_AGAP000529	-4.10	-3.35	-2.10	-2.49	-2.17	-1.78	-0.65
wmd	CG3957	STRAP	AgaP_AGAP005029	-4.56	-3.40	-1.60	-0.09	-0.10	-0.21	-0.06
yellow-c | l(2)35Bg	CG4182		AgaP_AGAP010280	2.85	2.18	2.21	0.86	1.01	0.43	-0.24
