# Supplementary material for: Genome-Wide RNAi Screen Identifies Broadly-Acting Host Factors That Inhibit Arbovirus Infection
Source: PLoS Pathog. 2014 Feb 13;10(2):e1003914. doi: 10.1371/journal.ppat.1003914 (PMC3923753; doi:10.1371/journal.ppat.1003914)
Supplement: Table S4 — List of VRFs identified in other screens. A compilation of genes identified in published screens with activity against flaviviruses are shown with those that have orthologs in Drosophila listed in red. Little overlap is observed. (PDF) [file ppat.1003914.s004.pdf]

TABLE S4: List of VRFs identified in other screens.
Genes in red have Drosophila orthologs.

Li ISG	Schoggins ISG	Li HCV	Krishnan WNV
ACSL4	APOL2	ARHGEF7	ACRC
AKAP8	BCL2L14	ATP11A	AMELY
ATF3	C15orf48	C16orf59	ANP32B
ATL1	C22orf28	CACNA1F	ATCAY
CDKN1A	C5orf39	COTL1	DEFA3
CXCR4	C6orf150	CSF3R	DHRS10
DDX24	CCDC109B	EMID2	DOCK4
EIF2AK2	CCL2	GKN2	FAM106A
GBP2	CCL8	HNRNPK	FKBP1B
GBP3	CD9	IGFBP6	IRF3
GNB4	CXCL11	IL17RB	KBTBD8
GPR126	DDX58	IMPDH1	KIAA0753
HRASLS2	ETV6	KCNA1	LPGAT1
HTR1D	FBXO6	KCNQ4	MSMB
ICAM1	GCH1	MTMR10	NFS1
IFI44L	GJA4	OTUB1	NPIP
IFI6	GK	PRPF31	NPM3
IFIT3	GZMB	PTPRH	SCGB2A1
IFITM2	HPSE	SEPT9	SERPINB7
IFRD1	HSH2D	SNRNP35	SLC16A4
IL13RA1	IFI16	TMPRSS12	SPTBN4
IL4I1	IFI6	TNFRSF18	TMEM146
IRF1	IFIH1	TSSK3	
IRF9	IFIT5	UBE2J2	
JAK2	IFITM3	USP43	
KBTBD8	IRF1		
KLK8	IRF2		
LPGAT1	IRF7		
MAFK	LRG1		
NUB1	MAB21L2		
PAK3	MKX		
PSMB9	P2RY6		
RGS22	PBEF1		
RNASE6	PHF15		
SAMD9	PI4K2B		
SAMD9L	PLSCR1		
SAMHD1	RTP4		
SC4MOL	SAA1		
SEMA6D	SLC1A1		
SERPINB7	SLFN5		
STAT2	TNFAIP6		
TLR3	TNFRSF10A		
TNFSF10	TNFSF13B		
TNFSF13B	TREX1		
TRIM21	TRIM25		
TRIM6	UNC93B		
VISA	ZBP1		
