# Supplementary material for: Genome-Wide RNAi Screen Identifies Broadly-Acting Host Factors That Inhibit Arbovirus Infection
Source: PLoS Pathog. 2014 Feb 13;10(2):e1003914. doi: 10.1371/journal.ppat.1003914 (PMC3923753; doi:10.1371/journal.ppat.1003914)
Supplement: Text S1 — Compilation of seven supplemental figures, their legends and associated methods are shown. (PDF) [file ppat.1003914.s005.pdf]

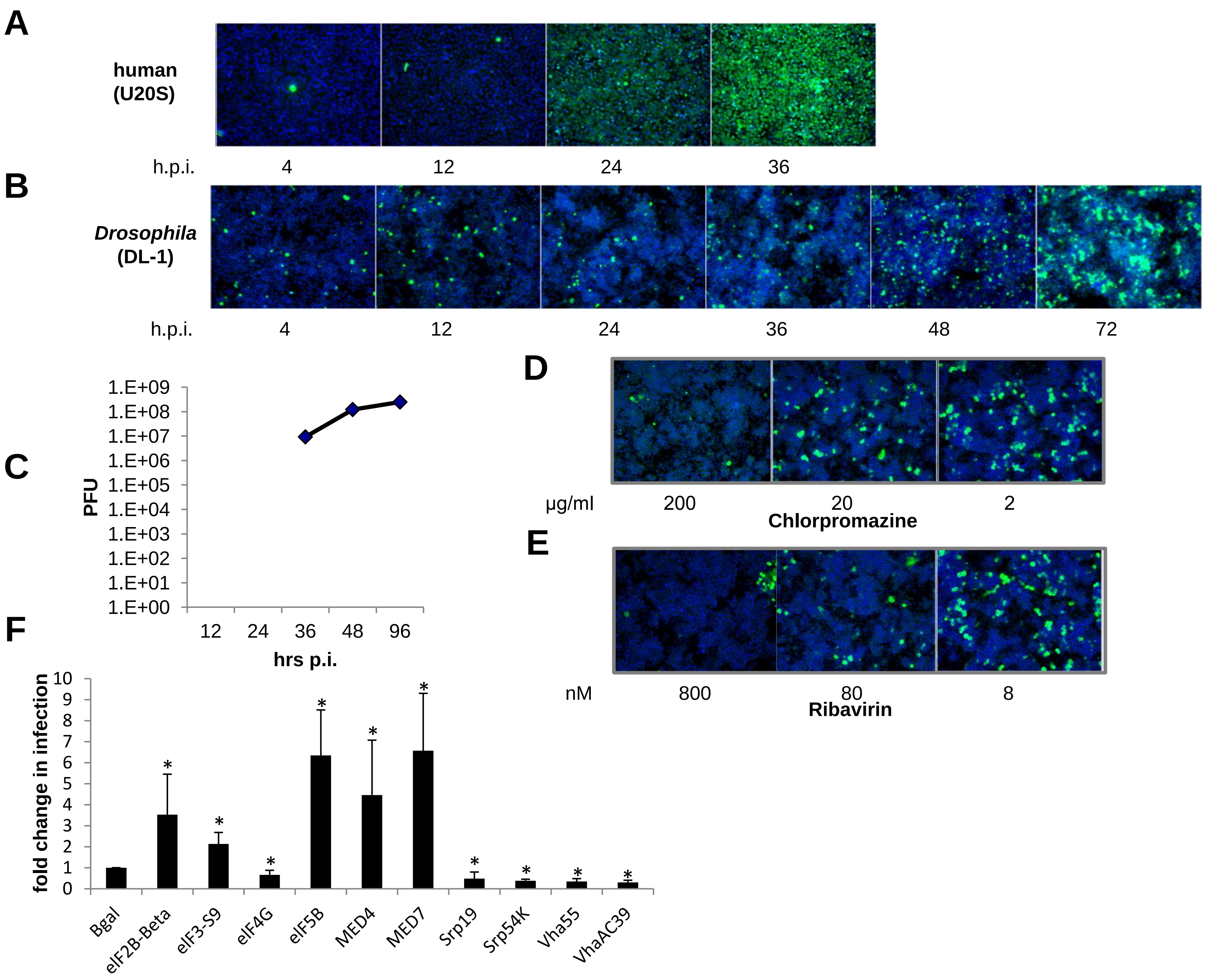
A
human
(U20S)
h.p.i.
4
12
24
36
B
Drosophila
(DL-1)
h.p.i.
4
12
24
36
48
72
C
PFU
1.E+09
1.E+08
1.E+07
1.E+06
1.E+05
1.E+04
1.E+03
1.E+02
1.E+01
1.E+00
12
24
36
48
96
hrs p.i.
D
µg/ml
200
20
2
Chlorpromazine
E
nM
800
80
8
Ribavirin
F
fold change in infection
10
9
8
7
6
5
4
3
2
1
0
*
Bgal
eIF2B-Beta
eIF3-S9
eIF4G
eIF5B
MED4
MED7
Srp19
Srp54K
Vha55
VhaAC39

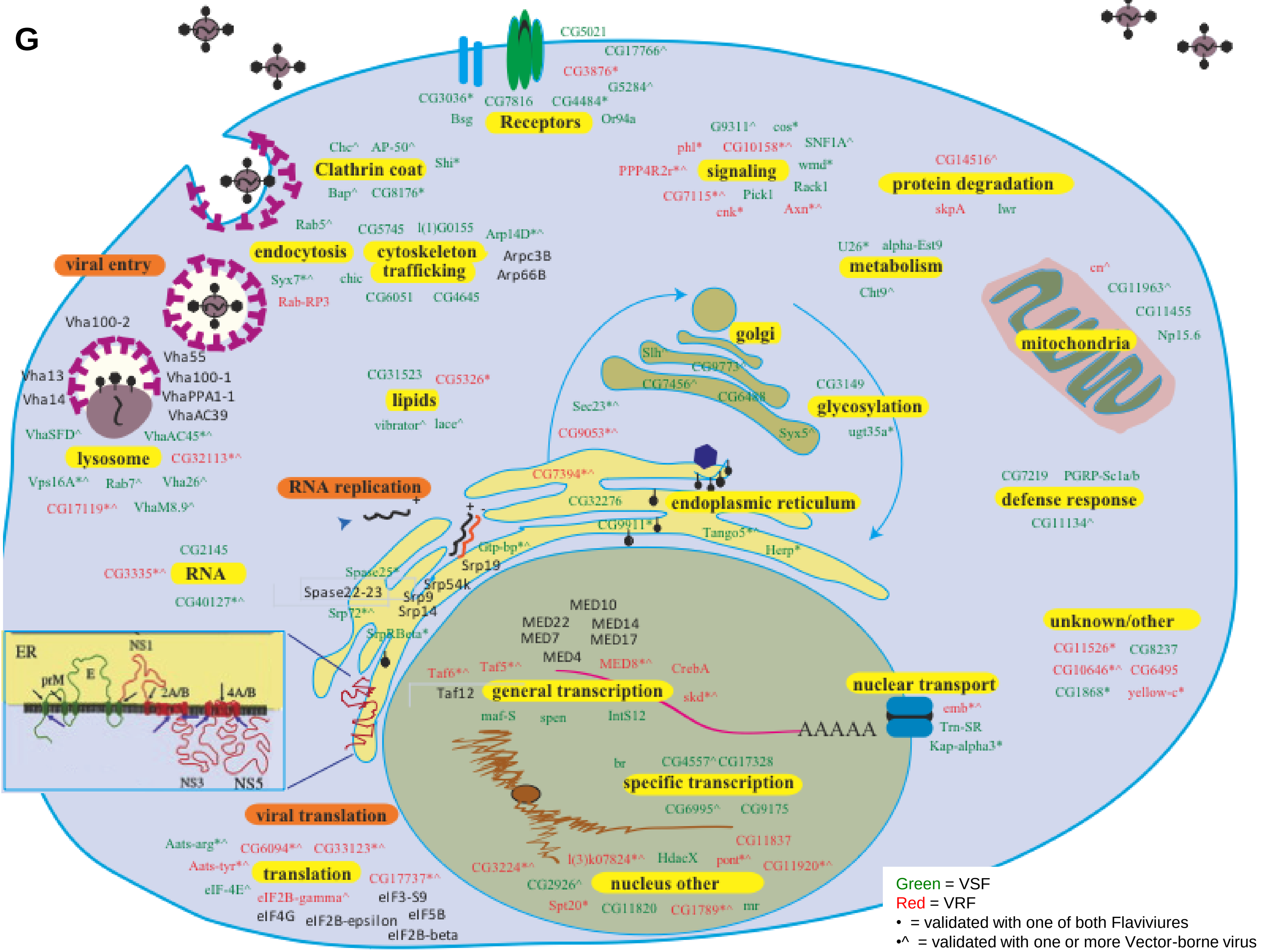
G
CG5021
CG17766^
CG3876*
G5284^
CG3036*
CG7816
CG4484*
Bsg
Receptors
Or94a
Chc^
AP-50^
Clathrin coat
Shi*
Bap^
CG8176*
G9311^
cos*
phl*
CG10158*^
SNF1A^
PPP4R2r*^
signaling
wmd*
Rack1
CG7115*^
Pick1
cnk*
Axn*^
CG14516^
protein degradation
skpA
lwr
Rab5^
CG5745
l(1)G0155
Arp14D*^
endocytosis
cytoskeleton trafficking
Arpc3B
Arp66B
viral entry
Syx7*^
chic
Rab-RP3
CG6051
CG4645
U26*
alpha-Est9
metabolism
Cht9^
cn^
CG11963^
CG11455
Np15.6
mitochondria
golgi
Slh
CG9773
CG7456^
CG6488
Syx5^
CG3149
glycosylation
ugt35a*
Vha100-2
Vha55
Vha13
Vha100-1
Vha14
VhaPPA1-1
VhaAC39
VhaSFD^
VhaAC45*^
lysosome
CG32113*^
Vps16A*^
Rab7^
Vha26^
CG17119*^
VhaM8.9^
CG31523
CG5326*
lipids
vibrator^
lace^
Sec23*^
CG9053*^
CG7394*^
CG32276
endoplasmic reticulum
CG9911*
Tango5*
Herp*
RNA replication
Gtp-bp*^
Srp19
CG7219
PGRP-Sc1a/b
defense response
CG11134^
CG2145
CG3335*^
RNA
CG40127*^
Spase25*
Spase22-23
Srp54k
Srp9
Srp72*^
Srp14
SrpRBeta*
MED10
MED22
MED14
MED7
MED17
MED4
MED8*^
CrebA
Taf6*^
Taf5*^
Taf12
general transcription
skd*^
maf-S
spen
IntS12
unknown/other
CG11526*
CG8237
CG10646*^
CG6495
CG1868*
yellow-c*
nuclear transport
emb*^
Trn-SR
Kap-alpha3*
AAAAA
ER
NS1
prM
E
2A/B
4A/B
NS3
NS5
br
CG4557^
CG17328
specific transcription
CG6995^
CG9175
viral translation
CG11837
Aats-arg*^
CG6094*^
CG33123*^
Aats-tyr*^
translation
CG17737*^
eIF-4E^
eIF2B-gamma^
eIF3-S9
eIF4G
eIF2B-epsilon
eIF5B
eIF2B-beta
CG3224*^
l(3)k07824*^
HdacX
pont*^
CG11920*^
CG2926^
nucleus other
Spt20*
CG11820
CG1789*^
mr
Green = VSF
Red = VRF
• = validated with one of both Flaviviures
•^ = validated with one or more Vector-borne virus

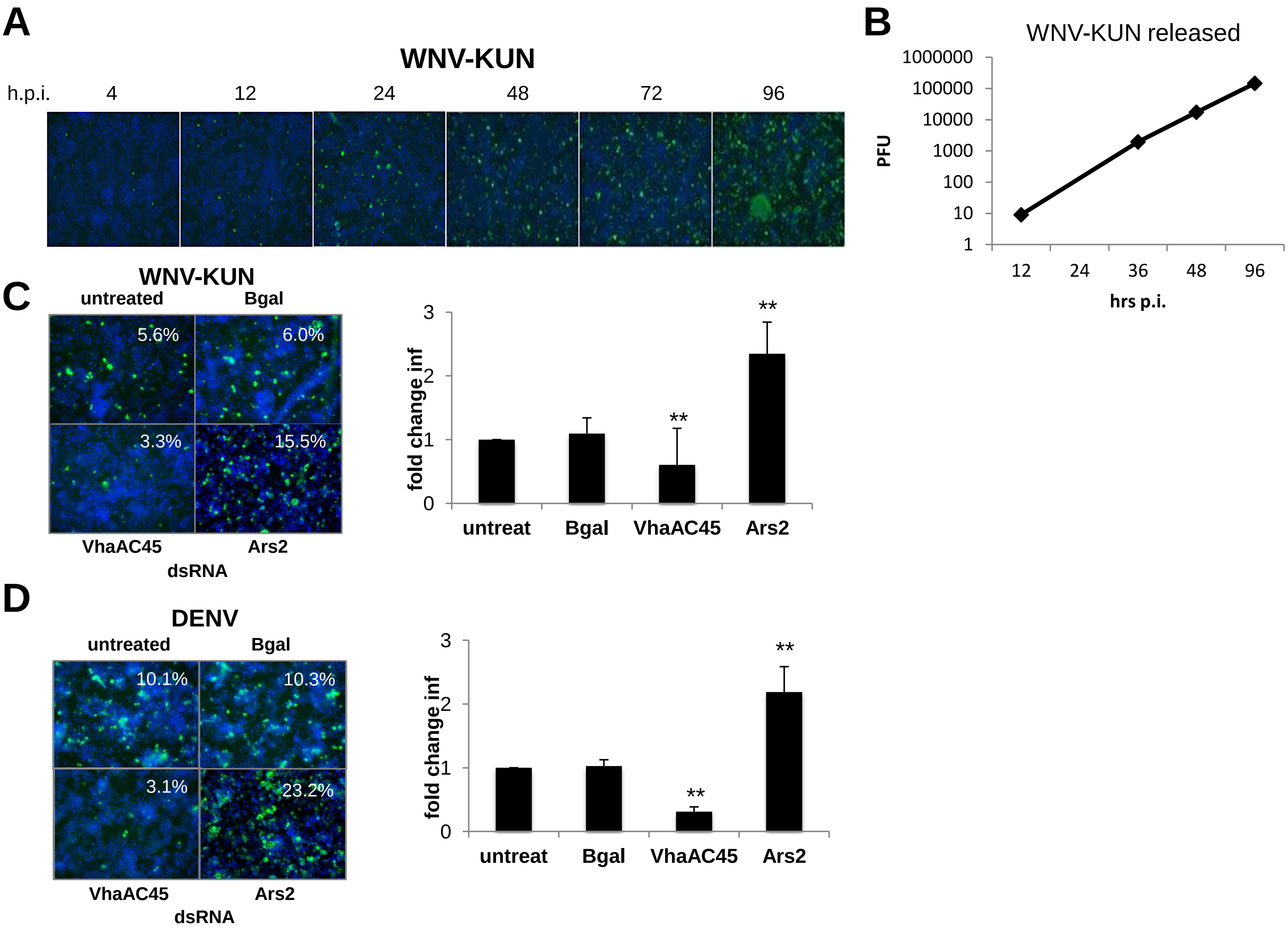
A
WNV-KUN
h.p.i.
4
12
24
48
72
96
B
WNV-KUN released
PFU
1000000
100000
10000
1000
100
10
1
12
24
36
48
96
hrs p.i.
C
WNV-KUN
untreated
Bgal
5.6%
6.0%
3.3%
15.5%
VhaAC45
Ars2
dsRNA
fold change inf
3
2
1
0
**
**
untreat
Bgal
VhaAC45
Ars2
D
DENV
untreated
Bgal
10.1%
10.3%
3.1%
23.2%
VhaAC45
Ars2
dsRNA
fold change inf
3
2
1
0
**
**
untreat
Bgal
VhaAC45
Ars2

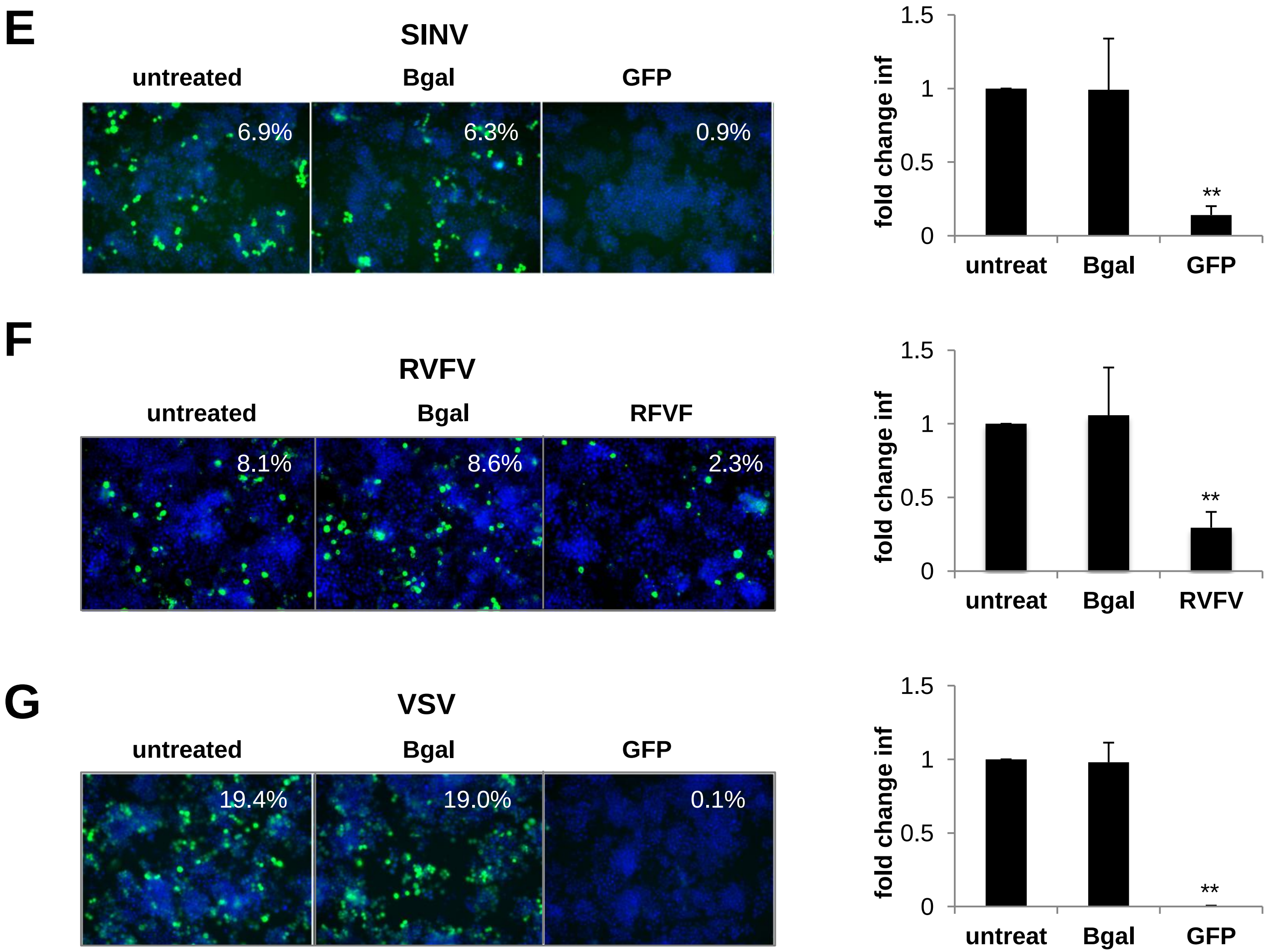
E
SINV
untreated
Bgal
GFP
6.9%
6.3%
0.9%
1.5
1
0.5
0
fold change inf
**
untreat
Bgal
GFP
F
RVFV
untreated
Bgal
RFVF
8.1%
8.6%
2.3%
1.5
1
0.5
0
fold change inf
**
untreat
Bgal
RVFV
G
VSV
untreated
Bgal
GFP
19.4%
19.0%
0.1%
1.5
1
0.5
0
fold change inf
**
untreat
Bgal
GFP

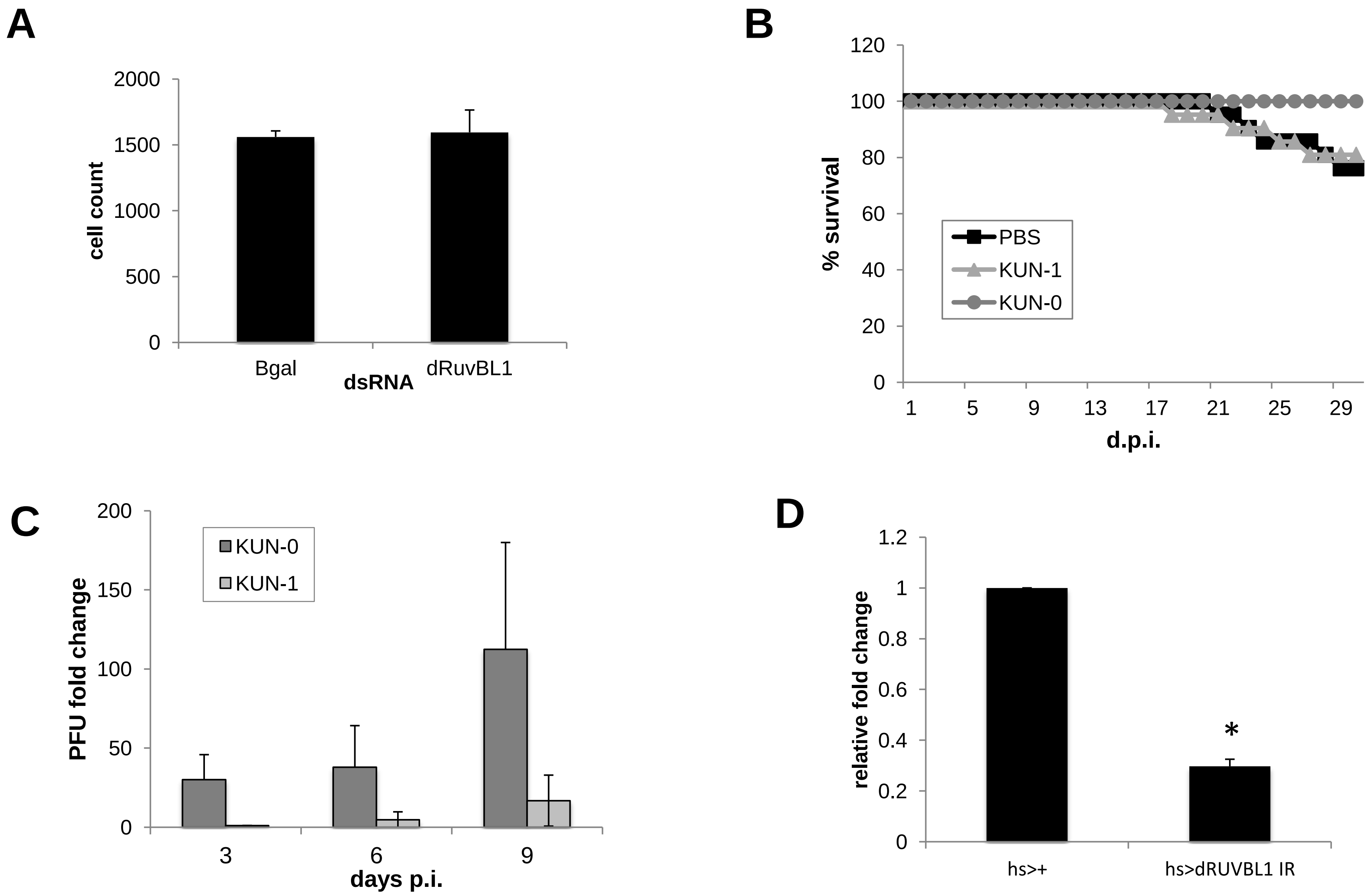
A
2000
1500
1000
500
0
cell count
Bgal
dRuvBL1
dsRNA
B
120
100
80
60
40
20
0
% survival
PBS
KUN-1
KUN-0
1
5
9
13
17
21
25
29
d.p.i.
C
200
150
100
50
0
PFU fold change
KUN-0
KUN-1
3
6
9
days p.i.
D
1.2
1
0.8
0.6
0.4
0.2
0
relative fold change
*
hs>+
hs>dRUVBL1 IR

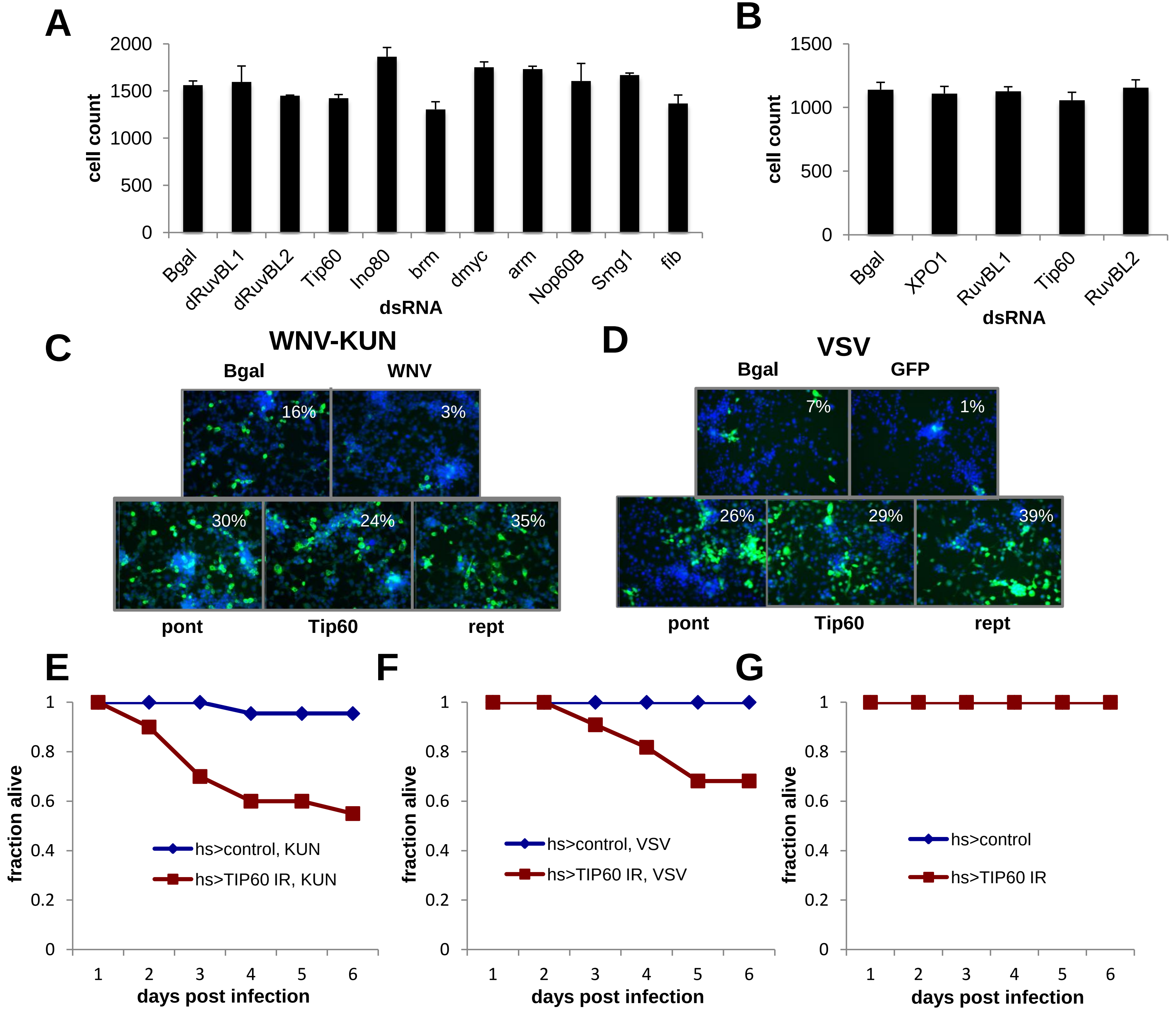
A
2000
1500
1000
500
0
cell count
Bgal
dRuvBL1
dRuvBL2
Tip60
Ino80
brm
dmyc
arm
Nop60B
Smg1
fib
dsRNA
B
1500
1000
500
0
cell count
Bgal
XPO1
RuvBL1
Tip60
RuvBL2
dsRNA
C
WNV-KUN
Bgal
WNV
16%
3%
30%
24%
35%
pont
Tip60
rept
D
VSV
Bgal
GFP
7%
1%
26%
29%
39%
pont
Tip60
rept
E
fraction alive
hs>control, KUN
hs>TIP60 IR, KUN
days post infection
F
fraction alive
hs>control, VSV
hs>TIP60 IR, VSV
days post infection
G
fraction alive
hs>control
hs>TIP60 IR
days post infection

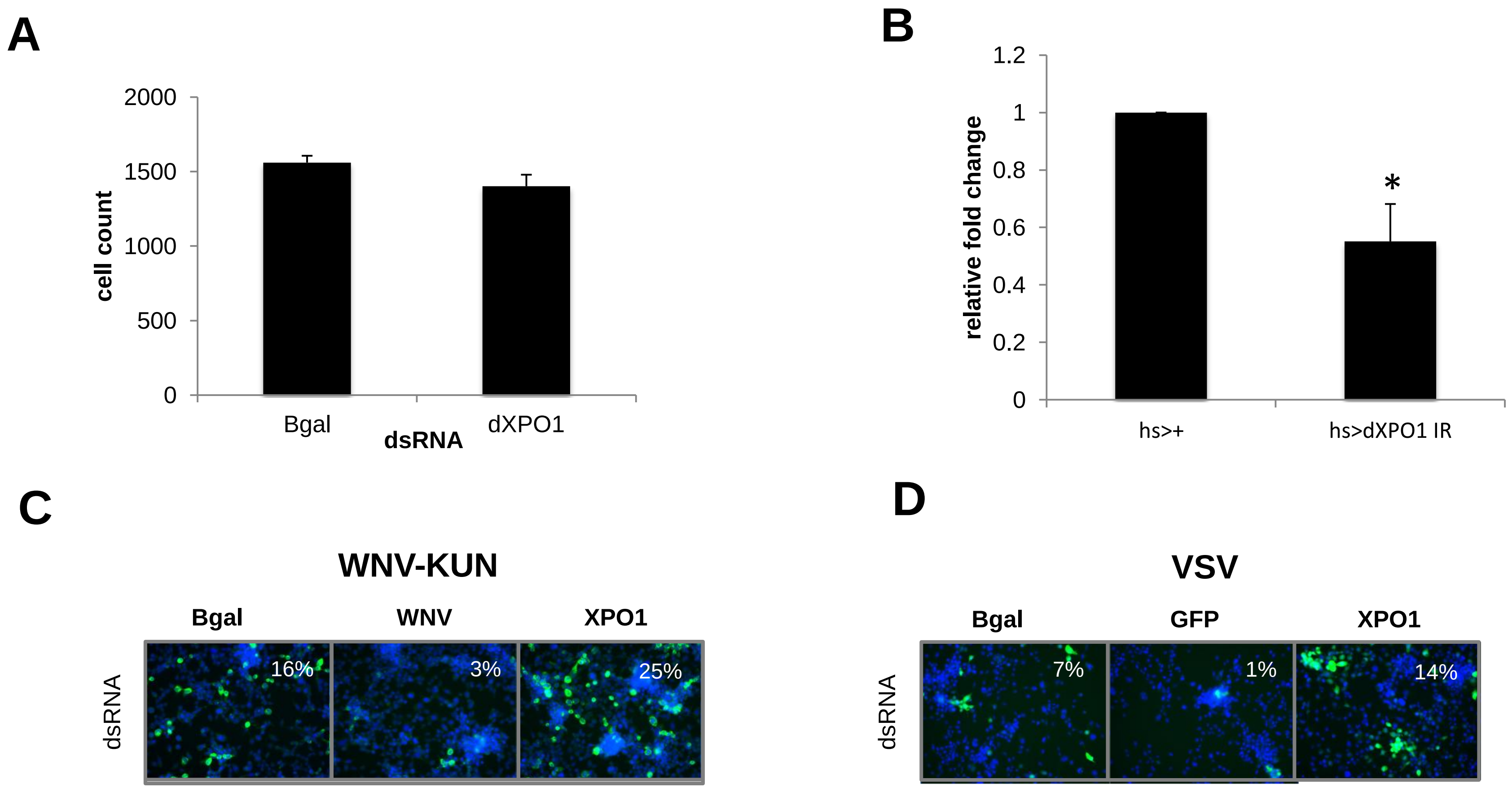
A
2000
1500
1000
500
0
cell count
Bgal
dXPO1
dsRNA
B
1.2
1
0.8
0.6
0.4
0.2
0
relative fold change
*
hs>+
hs>dXPO1 IR
C
WNV-KUN
Bgal
WNV
XPO1
dsRNA
16%
3%
25%
D
VSV
Bgal
GFP
XPO1
dsRNA
7%
1%
14%

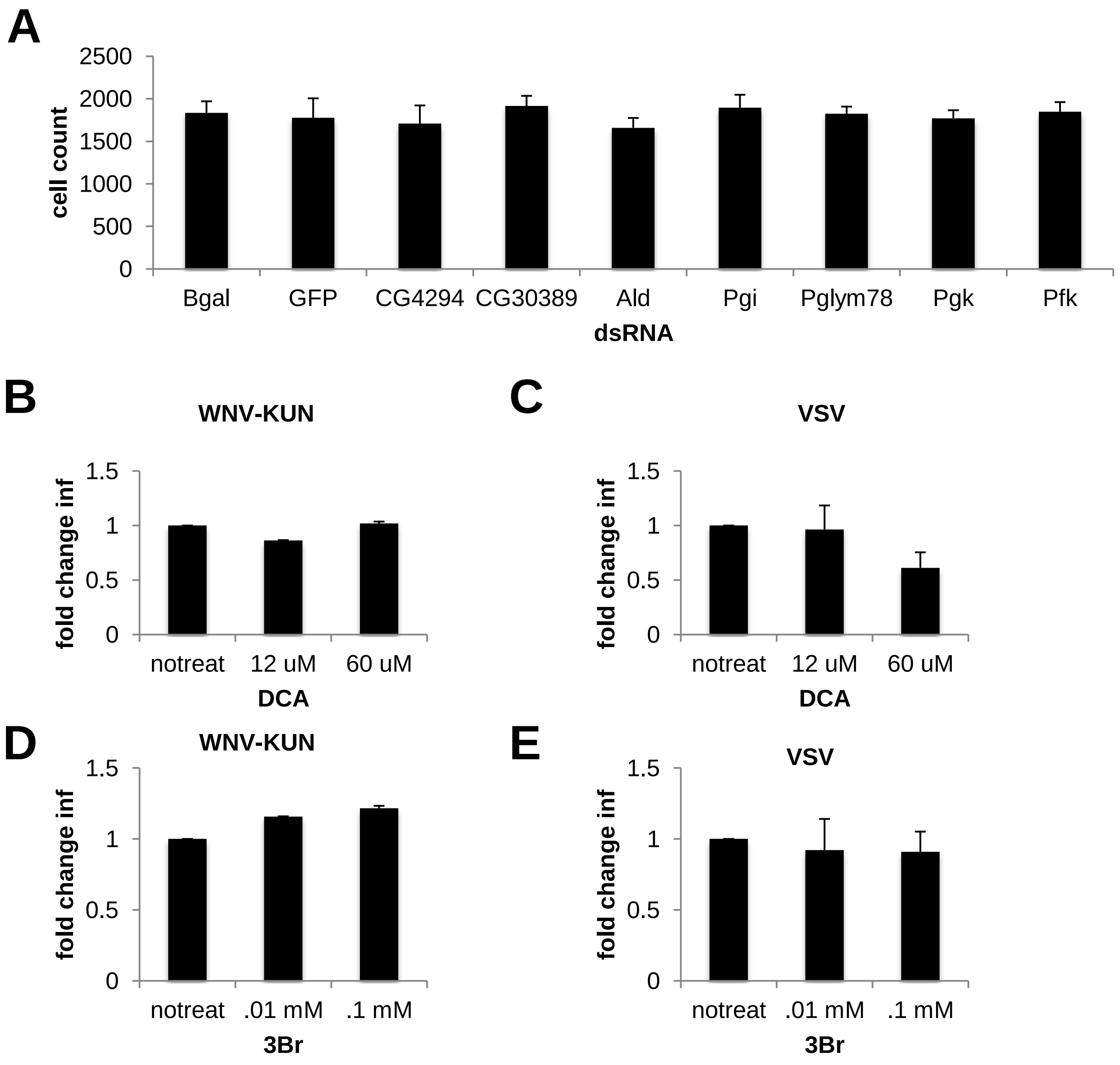

A
cell count
2500
2000
1500
1000
500
0
Bgal
GFP
CG4294
CG30389
Ald
Pgi
Pglym78
Pgk
Pfk
dsRNA
B
WNV-KUN
fold change inf
1.5
1
0.5
0
notreat
12 uM
60 uM
DCA
C
VSV
fold change inf
1.5
1
0.5
0
notreat
12 uM
60 uM
DCA
D
WNV-KUN
fold change inf
1.5
1
0.5
0
notreat
.01 mM
.1 mM
3Br
E
VSV
fold change inf
1.5
1
0.5
0
notreat
.01 mM
.1 mM
3Br

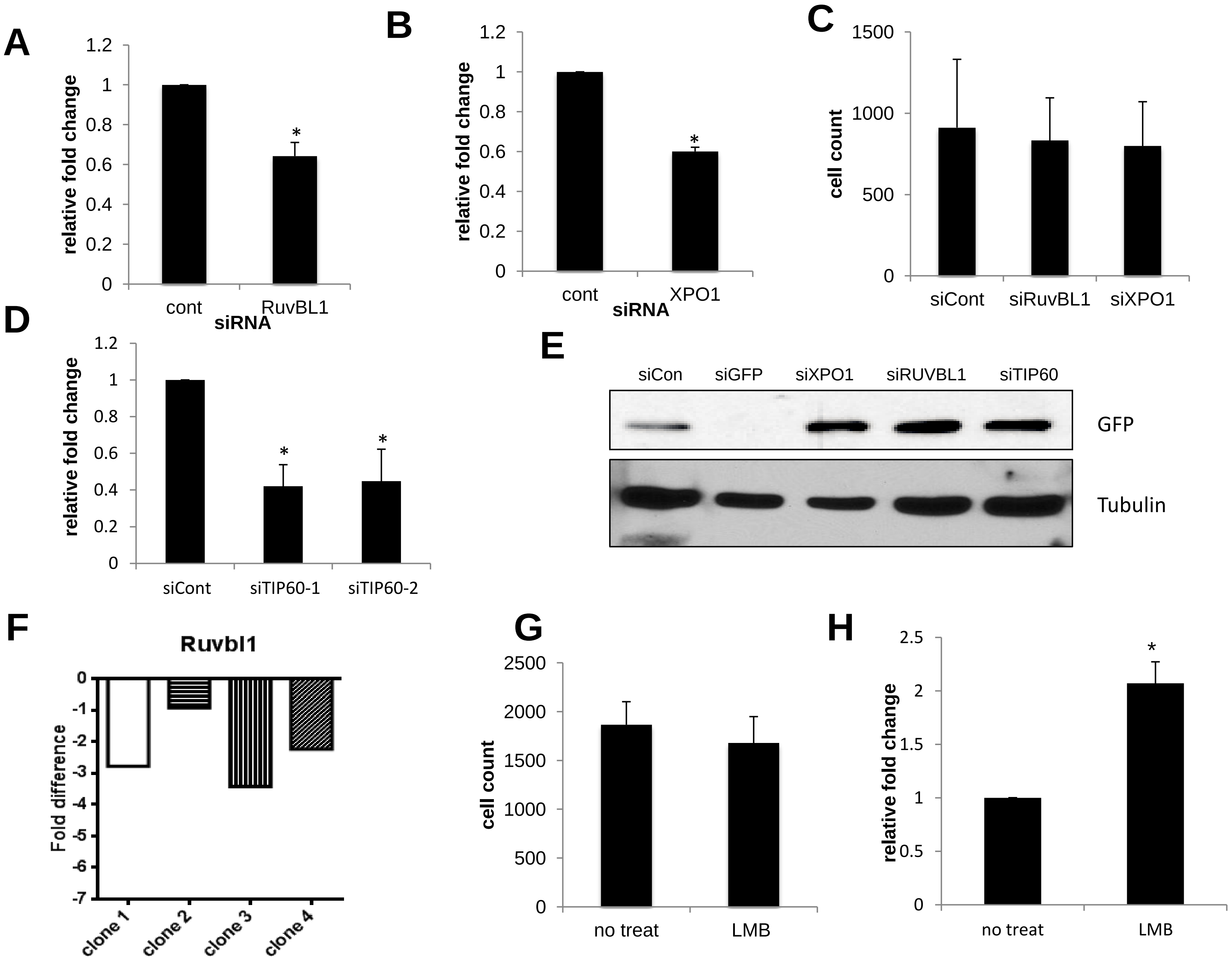

A
1.2
1
0.8
0.6
0.4
0.2
0
relative fold change
*
cont
RuvBL1
siRNA
B
1.2
1
0.8
0.6
0.4
0.2
0
relative fold change
*
cont
XPO1
siRNA
C
1500
1000
500
0
cell count
siCont
siRuvBL1
siXPO1
D
1.2
1
0.8
0.6
0.4
0.2
0
relative fold change
*
*
siCont
siTIP60-1
siTIP60-2
E
siCon
siGFP
siXPO1
siRUVBL1
siTIP60
GFP
Tubulin
F
Ruvbl1
0
-1
-2
-3
-4
-5
-6
-7
Fold difference
clone 1
clone 2
clone 3
clone 4
G
2500
2000
1500
1000
500
0
cell count
no treat
LMB
H
2.5
2
1.5
1
0.5
0
relative fold change
*
no treat
LMB

Supplemental Figures.

Figure S1. Characterization of WNV infection and schematic of validated screening results.

A-B. Images of WNV-infected (**A**) human U20S cells (MOI 0.5) or (**B**) *Drosophila* cells (MOI 10) that were fixed at indicated time points post infection ((blue, nuclei; green, virus). **C.** Supernatant from WNV-infected DL1 cells collected at designated time points post infection was titered on BHK cells to determine infectious viral release. Initial viral inoculum was removed after 12 hrs, cells washed of residual virus, and fresh media replaced. **D-E.** Representative images of DL1 cells pretreated with (**D**) chlorpromazine or (**E**) ribavirin at designated concentrations for 30 minutes prior to infection with WNV (MOI 10) (blue, nuclei; green, virus). **F**. *Drosophila* cells were treated with the indicated dsRNAs for three days and infected with WNV-KUN (MOI of 10) for 48 hours and processed for microscopy. Quantification of the fold change in infection is presented as mean ± SD for three independent experiments; * $p<0.05$. **G.** Pictorial schematic of a cell detailing the predominant cellular function of validated genes identified in the WNV genome-wide RNAi screen. VSFs and VRFs confirmed in the secondary screen are identified in green and red respectively. Those genes included as part of a validated complex are in black. * identifies genes also validated with one or more of the other vector-borne viruses tested. Underlined genes impacted infection with all the viruses tested.

Figure S2. Optimization of arbovirus screening assay.

A. Representative images of WNV-KUN-infected DL1 cells (MOI 10) that were fixed at 4, 12, 24, 48 and 96 hrs post infection (blue, nuclei; green, virus). **B.** Supernatant from WNV-KUN-infected DL1 cells collected at designated time points post infection and titered on BHK cells to determine infectious virus release. Initial viral inoculum was removed after 12 hrs, cells washed of residual virus, and fresh media replaced. **C-G,** Left, representative images of dsRNA-treated DL1 cells infected with (**C**) WNV-KUN, (**D**) DENV, (**E**) SINV, (**F**) RVFV, or (**G**) VSV. Right, Mean ± SD of fold change in infection normalized to untreated for 3 independent experiments; ** $p<0.01$.

Figure S3. Characterization of WNV-KUN infection of flies.

A. Gene silencing of dRUVBL1 does not impact cell viability as measured by nuclei number. Mean ± SD for 3 independent experiments. **B-C**. Adult wild-type flies (w1118) were challenged with WNV-KUN at two different dilutions (100 PFU (KUN-0) or 10 PFU (KUN-1)) and (**B**) mortality was monitored with a representative experiment shown; similar findings were made in at least three experiments. (**C**) Viral titer was monitored at the indicated day post infection. Results of at least 3 experiments are plotted as Mean ± SD in the fold change in PFU setting D3 KUN-1 to one. **D**. RT-qPCR was used to confirm knockdown of dRUVBL1 in hs>dRUVBL1 IR heat shocked flies. Mean ± SD of fold change normalized to control for 3 independent experiments; * $p<0.05$.

Figure S4. Tip60 complex is antiviral in insects.
A-B. Silencing of indicated genes in (**A**) DL1 or (**B**) Aag2 cells does not impact cell number. Mean ± SD of cell number normalized to control dsRNA (bgal) for 3 independent experiments. **C-D**. Representative images of dsRNA-treated Aag2 cells infected with (**C**) WNV-KUN, (**D**) VSV. **E-G**. Adult flies of the indicated genotypes were challenged with WNV-KUN (**E)** VSV **(F**) or vehicle (**G)**. Mortality was monitored as a function of time post-infection.

Figure S5. XPO1 is antiviral in insects
A. Silencing of dXPO1 does not impact cell viability as measured by nuclei number. Mean ± SD of cell number normalized to control dsRNA (bgal) for 3 independent experiments. **B**. qRT-PCR was used to confirm knockdown of dXPO1 in hs>dXPO1 IR heat shocked flies. Mean ± SD of fold change normalized to control for 3 independent experiments; * $p<0.05$. **C-D**. Representative images of dsRNA-treated Aag2 cells infected with (**C**) WNV-KUN or (**D**) VSV.

Figure S6. Glycolysis does not control infection.
A. Silencing of indicated genes does not impact cell viability as measured by nuclei number. Mean ± SD of cell number normalized to control dsRNA (bgal) for 3 independent experiments. **B-E.** DL1 cells pretreated with (**B-C)** DCA or (**D-E**) 3Br at indicated concentrations 30 minutes prior to infection compared to untreated cells. Mean ± SD for fold

change in infection normalized to control (notreat) shown for three independent experiments. **B,D**, WNV-KUN infected. **C,E,** VSV infected.

Figure S7. RUVBL1 and XPO1 are antiviral in human cells.

A-C, Human U2OS cells were transfected with indicated siRNAs. 3 days post transfection qRT-PCR was used to confirm knockdown with (**A**) hRUVBL1 siRNA or (**B**) hXPO1 siRNA. **C**. Cell numbers were not affected. Mean ± SD for 3 independent experiments; * $p<0.05$. **D**. Human 293T cells were transfected with siRNAs against TIP60 and monitored for TIP60 levels by RT-qPCR. Mean ± SD for 3 independent experiments; * $p<0.05$. **E.** 293T cells expressing a subgenomic WNV replicon (expressing GFP) were transfected with the indicated siRNAs for four days and analyzed by immunoblot. A representative blot is shown. **F.** Mouse neurons were infected with the indicated lentiviruses expressing one of four shRNAs against RUVBL1 and qRT-PCR was used to confirm knockdown. **G.** There was no decrease in viability upon treatment with LMB in U2OS cells as measured by cell number. Mean ± SD shown for three independent experiments. **H.** 293T cells expressing a subgenomic WNV replicon (expressing GFP) were treated with LMB and analyzed by microscopy. Mean ± SD of GFP shown normalized to untreated cells; * $p<0.05$.

Supplementary Methods

Gene silencing validation:

To determine efficiency of gene silencing in flies, total RNA was isolated from 15 flies of the indicated genotypes. cDNA was generated using random hexamers and MLV-RT (Invitrogen). Transcripts were quantified using Sybrgreen with the primers (dRUVBL1: Forward CTTCACATTTCCTCTCCCTCG; Reverse GCCTCTAAATCCGTGTCCTG
and dXPO1: Forward TGCTGGAGAACTCGATGAATG; Reverse GGAACATGGGTACGCTAAGG). Cellular levels of rp49 were quantified in parallel and used for normalization.

To determine efficiency of gene silencing in neurons, total RNA was isolated from shRNA-transduced cerebellar granule cell neurons. cDNA was generated from RNA using random hexamers and Multiscribe reverse transcriptase (Applied Biosystems). Transcripts were quantified on a 7500 Fast Real-time PCR system (Applied Biosystems) with Taqman primers and probe sets (Forward

ATGACCTGGATGTGGCTAATG; Reverse CTCCCCTCGAAGTTTATCTGTG; and Probe CCTCAGGGTGGGCAAGATATTCTGTC). Cellular levels of 18S ribosomal RNA were quantified in parallel by TaqMan analysis and used for normalization.
